# Supplementary material for: A third dose of the unmodified COVID-19 mRNA vaccine CVnCoV enhances quality and quantity of immune responses
Source: Mol Ther Methods Clin Dev. 2022 Oct 6;27:309–23. doi: 10.1016/j.omtm.2022.10.001 (PMC9535876; doi:10.1016/j.omtm.2022.10.001)
Supplement: Document S1. Figures S1–S4 and Table S1 — –S4 [file mmc1.pdf]

OMTM, Volume *27*

Supplemental information

A third dose of the unmodified COVID-19 mRNA vaccine CVnCoV enhances quality and quantity of immune responses

Klara Lenart, Fredrika Hellgren, Sebastian Ols, Xianglei Yan, Alberto Cagigi, Rodrigo Arcoverde Cerveira, Inga Winge, Jakub Hanczak, Stefan O. Mueller, Edith Jasny, Kim Schwendt, Susanne Rauch, Benjamin Petsch, and Karin Loré

SUPPLEMENTAL MATERIALS

SUPPLEMENTAL FIGURES

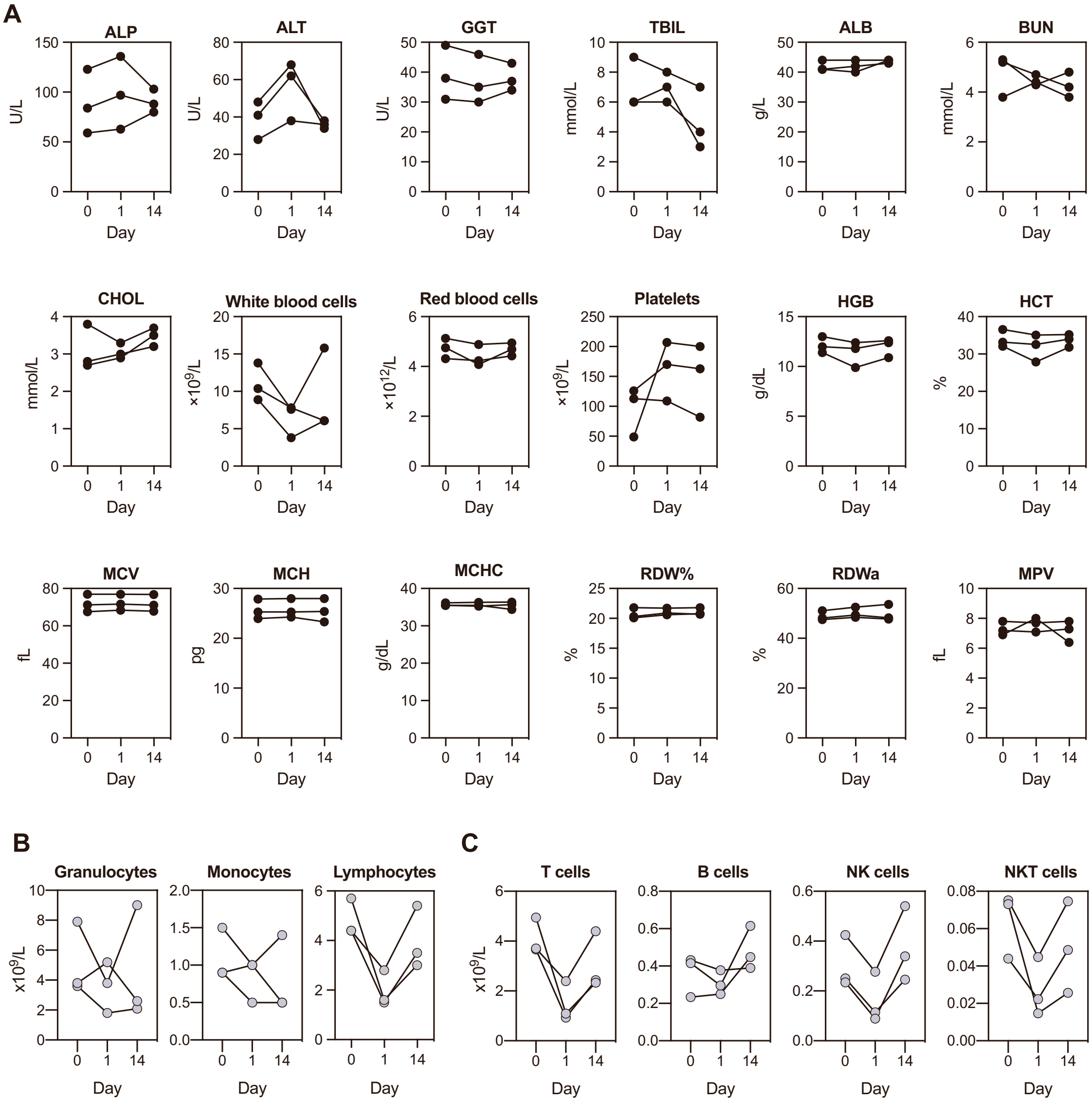

Figure S1: **Safety and innate data after prime mRNA immunization.** (A) Clinical chemistry and hematology analyses at 0, 1 and 14 days after prime immunization. (B) Complete blood counts (CBCs) performed at 0, 1 and 14 days after prime immunization. (C) Immune cell subsets quantified by flow cytometry and normalized to lymphocyte CBC data at days 0, 1 and 14 after prime immunization. ALP = Alkaline Phosphatase, ALT = Alanine Aminotransferase, GGT = Gamma-Glutamyl Transferase, TBIL = Total Bilirubin, ALB = Albumin, BUN = Blood Urea Nitrogen, CHOL = Cholesterol, HGB = Hemoglobin, HCT = Hematocrit, MCV = Mean Corpuscular Volume, MCH = Mean Corpuscular Hemoglobin, MCHC = Mean Corpuscular Hemoglobin Concentration, RDW% = Red Cell Distribution, RDWa = Red Cell Distribution (Absolute), MPV = Mean Platelet Volume.

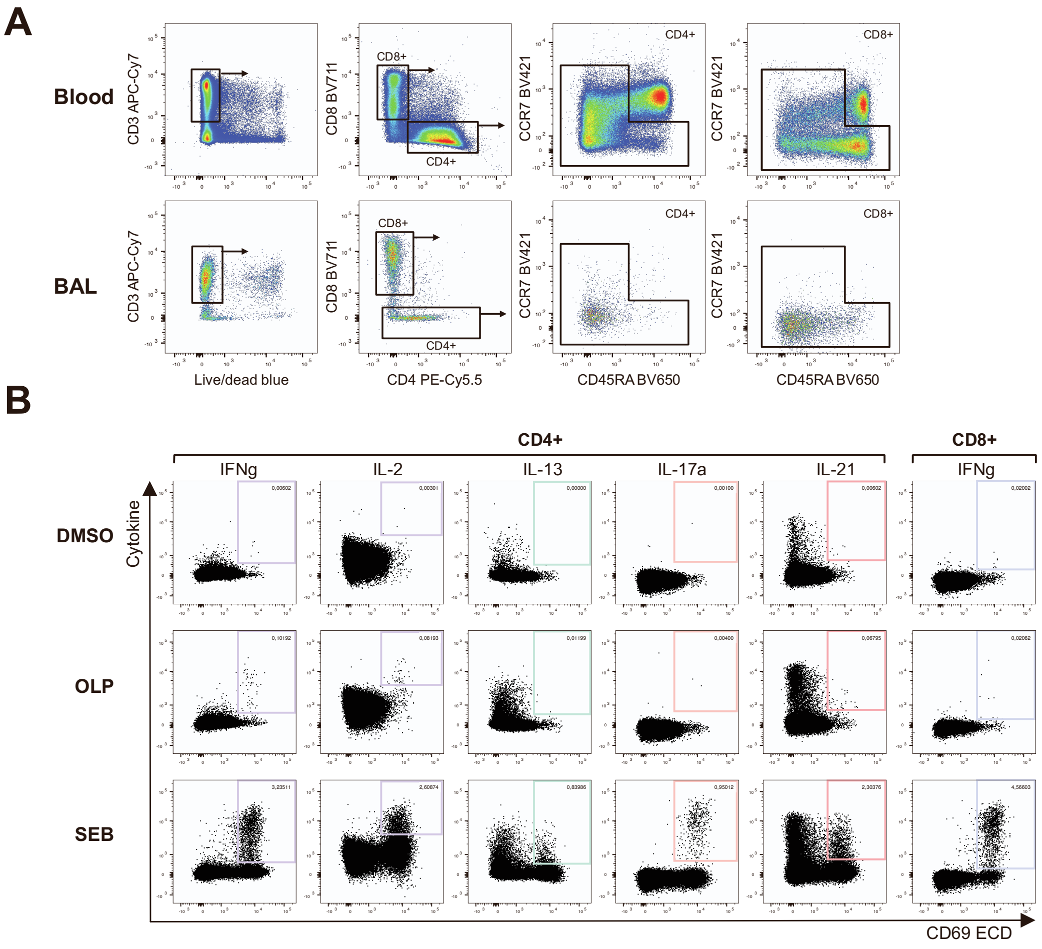

Figure S2. (A) Gating strategy used to define CD4+ and CD8+ T cells in blood and BAL. (B) Definition of responding antigen-specific T cells by intracellular cytokine staining.

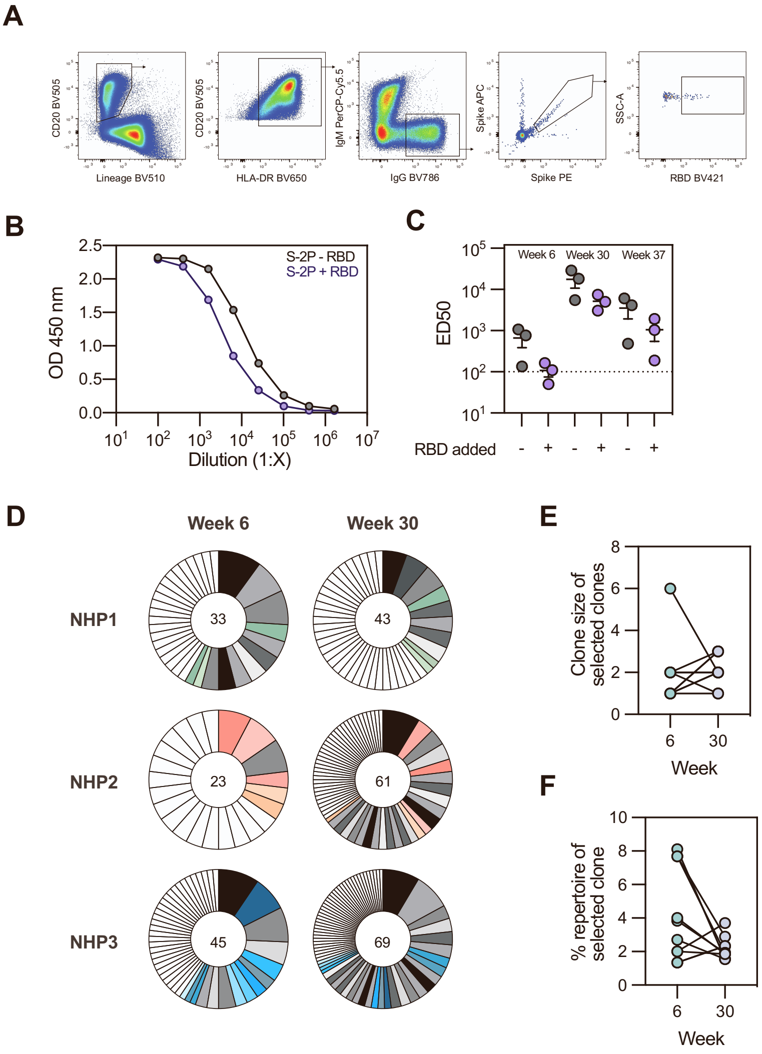

Figure S3. (A) Gating strategy used to define antigen-specific memory B cells. Lineage channel contains CD3, CD11c, CD14, CD16 and CD123. (B, C) ELISA curves (B) and ED50 values (C) of NHP sera with and without soluble RBD as competitor, used to calculate proportion of RBD-binding antibodies. (D) Clonotyping of sorted Spike-specific memory B cells. Each pie chart represents the sequenced repertoire in a NHP at a selected timepoint. The number in the middle indicates number of clones. Pie slice size is proportional to the clone expansion at a given timepoint. Colored pie slices represent clones detected at both analyzed timepoints. (E, F) Sizes of clones, from which selected mAbs were derived from, plotted as absolute number of sequences in the clone (E) and as percentage of repertoire sequenced (F).

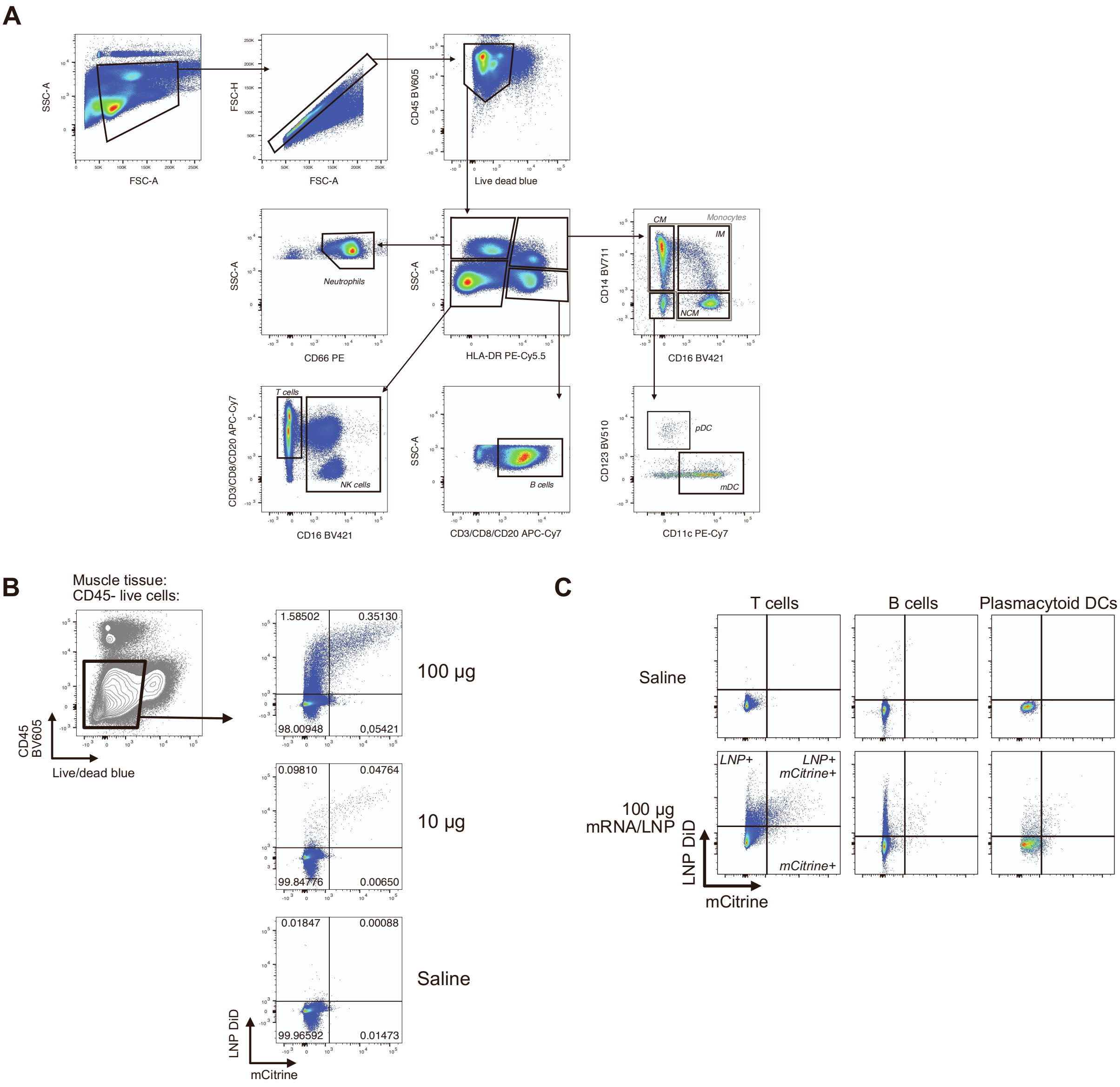

Figure S4. (A) Gating strategy used to define immune cell subsets in blood and tissues after mRNA vaccine immunization in biodistribution experiments. (B) LNP uptake and mRNA vaccine-derived protein expression in non-immune cells at site of injection (muscle tissue). (C) Representative flow cytometry plots of LNP DiD and mCitrine signal in draining lymph nodes by cell subset (T cells, B cell and plasmacytoid DCs) from saline- and vaccine-injected sites.

SUPPLEMENTAL TABLES

Table S1: Innate immunophenotyping antibody panel

Antibody	Clone	Manufacturer
CD40-FITC	5C3	Biolegend
NKg2a-PE	Z199	Beckman Coulter
CD80-BV421	L307.4	BD
CCR7-PE-Dazzle 594	G043H7	Biolegend
CD123-PerCp-Cy5.5	7G3	BD
CD3-APC-Cy7	SP34-2	BD
CD66-APC	TET2	Miltenyi
CD70-BV786	Ki-24	BD
HLA-DR-BV650	L243	Biolegend
CD11c-PE-Cy7	3.9	Biolegend
CD16-AF700	38G	BD
CD20-BV605	2H7	Biolegend
CD14-BV510	M5E2	Biolegend

Table S2: T cell ICS staining antibody panel (*Intracellular staining)

Antibody	Clone	Manufacturer
CD103-FITC	2G5	Beckman
CCR7-BV421	G043H7	Biolegend
CD8a-BV711	RPA-T8	Biolegend
CD4-PE-Cy55	S3.5	Invitrogen
CD45RA-BV650	5H9	BD
*IL-21-AF647	3A3-N2.1	BD
*IL-13-PE	JES10-5A2	BD
*IL-2-BV605	MQ1-17H12	BD
*IL-17A-BV785	BL168	Biolegend
*CD69-ECD	TP1.55.3	Beckman
*CD3-APC-Cy7	SP34.2	BD
*IFNg-AF700	B27	Biolegend

Table S3: Memory B cell antibody panel

Antibody	Clone	Manufacturer
7AAD	NA	Thermo Fisher
IgM-PerCP-Cy5.5	G20-127	BD
CD3-BV510	SP34-2	BD
CD123-BV510	6H6	Biolegend
CD11c-BV510	3.9	Biolegend
CD16-BV510	3G8	BD
HLA-DR-BV650	L243	Biolegend
IgG-BV786	G18-145	BD
CD20-BV605	2H7	Biolegend
CD14-BV510	M5E2	Biolegend

Table S4: Biodistribution antibody panel (*NHP samples only)

Antibody	Clone	Manufacturer
CD66abce-PE	TET2	Miltenyi
CD11c-PE-Cy7	3.9	Biolegend
CD14-BV711	M5E2	Biolegend
CD123-BV510	6H6	Biolegend
CD3-APC-Cy7	SP34-2	BD
CD20-APC-Cy7	L27	BD
CD8-APC-Cy7	RPA-T8	Biolegend
CD80-BV650	L307.4	BD
*CD45-BV605	D058-1283	BD
HLA-DR-PE-Cy55	Tu36	Life Tech
CD16-BV421	3G8	Biolegend
