# Supplementary material for: A third dose of the unmodified COVID-19 mRNA vaccine CVnCoV enhances quality and quantity of immune responses
Source: Mol Ther Methods Clin Dev. 2022 Oct 6;27:309–23. doi: 10.1016/j.omtm.2022.10.001 (PMC9535876; doi:10.1016/j.omtm.2022.10.001)
Supplement: Document S2. Article plus supplemental information [file mmc2.pdf]

Molecular Therapy
Methods & Clinical Development
Original Article

A third dose of the unmodified COVID-19 mRNA vaccine CVnCoV enhances quality and quantity of immune responses

Klara Lenart,[1,2] Fredrika Hellgren,[1,2] Sebastian Ols,[1,2] Xianglei Yan,[1,2] Alberto Cagigi,[1,2] Rodrigo Arcoverde Cerveira,[1,2] Inga Winge,[1,2] Jakub Hanczak,[1,2] Stefan O. Mueller,[3] Edith Jasny,[3] Kim Schwendt,[3] Susanne Rauch,[3] Benjamin Petsch,[3] and Karin Loré[1,2]

[1]Department of Medicine Solna, Division of Immunology and Allergy, Karolinska Institutet and Karolinska University Hospital, Stockholm, Sweden; [2]Center for Molecular Medicine, Karolinska Institutet, Stockholm, Sweden; [3]CureVac AG, Tübingen, Germany

A third vaccine dose is often required to achieve potent, long-lasting immune responses. We investigated the effect of three 8-µg doses of CVnCoV, CureVac's severe acute respiratory syndrome coronavirus 2 (SARS-CoV-2) vaccine candidate containing sequence-optimized unmodified mRNA encoding the spike (S) glycoprotein, administered at 0, 4, and 28 weeks, on immune responses in rhesus macaques. After the third dose, S-specific binding and neutralizing antibodies increased 50-fold compared with post-dose 2 levels, with increased responses also evident in the lower airways and against the SARS-CoV-2 B.1.1.7 (Alpha), B.1.351 (Beta), P.1 (Gamma), and B.1.617.2 (Delta) variants. Enhanced binding affinity of serum antibodies after the third dose correlated with higher somatic hypermutation in S-specific B cells, corresponding with improved binding properties of monoclonal antibodies expressed from isolated B cells. Administration of low-dose mRNA led to fewer cells expressing antigen *in vivo* at the injection site and in the draining lymph nodes compared with a 10-fold higher dose, possibly reducing engagement of precursor cells with the antigen and resulting in the suboptimal response observed after two-dose vaccination schedules in phase IIb/III clinical trials of CVnCoV. However, when immune memory is established, a third dose efficiently boosts the immunological responses and improves antibody affinity and breadth.

INTRODUCTION

The coronavirus disease 2019 (COVID-19) pandemic resulted in accelerated development of vaccines against severe acute respiratory syndrome coronavirus 2 (SARS-CoV-2), with 38 approved vaccines in 197 countries and 212 candidates in testing as of June 2022.[1] The most notable of these were two nucleoside-modified mRNA vaccines (BNT162b2 [BioNTech/Pfizer] and mRNA-1273 [Moderna]) that were rapidly authorized, manufactured, and distributed, whereas several other sequence-optimized, chemically unmodified mRNA vaccines are still in clinical development.[1] CureVac's vaccine candidate, CVnCoV, was the first unmodified mRNA SARS-CoV-2 vaccine to reach phase III clinical testing.[2] In the reported clinical trials of CVnCoV, much lower doses of unmodified mRNA (2–12 µg) were tested than those used in modified mRNA vaccines; e.g., 30 µg in the BioNTech/Pfizer vaccine[3] and 100 µg in the Moderna vaccine.[4] This was because unmodified mRNA vaccines are considered to induce stronger innate immune activation, which, at high doses, may lead to reactogenicity. A trend of a dose-dependent increase in local and systemic solicited events was observed in the phase 1 dose-escalation study of CVnCoV.[5]

In the phase IIb/III HERALD trial, the CVnCoV vaccine candidate, containing 12 µg mRNA encapsulated in lipid nanoparticles (LNPs), showed an overall vaccine efficacy (VE) of 48.2% against COVID-19 of any severity and 70.7% against moderate to severe COVID-19, measured in an environment with 15 different circulating SARS-CoV-2 variants.[2] CureVac decided to discontinue development of CVnCoV and focus on new-generation candidates.[6]

To inform the development of better strategies for immunization with more efficacious vaccine candidates, we performed a detailed immunological investigation to understand the magnitude and quality of the immune responses to CVnCoV, including an assessment of the effect of a third 8-µg dose of CVnCoV on the immune response in non-human primates (NHPs). The NHP model allowed us to take multiple samples over time and to collect not only blood samples but also respiratory samples for assessment of mucosal responses and bone marrow for long-lived plasma cell responses. In a two-dose vaccination schedule, 8 µg CVnCoV has been shown to induce seroconversion but with relatively low antibody titers in humans[5] and

Received 4 August 2022; accepted 4 October 2022;
https://doi.org/10.1016/j.omtm.2022.10.001.

Correspondence: Karin Loré, Division of Immunology and Allergy, Department of Medicine Solna, Karolinska Institutet, Visionsgatan 4, BioClinicum J7:30, Karolinska University Hospital, 171 64 Stockholm, Sweden.
E-mail: karin.lore@ki.se

This is an open access article under the CC BY license (http://creativecommons.org/licenses/by/4.0/).

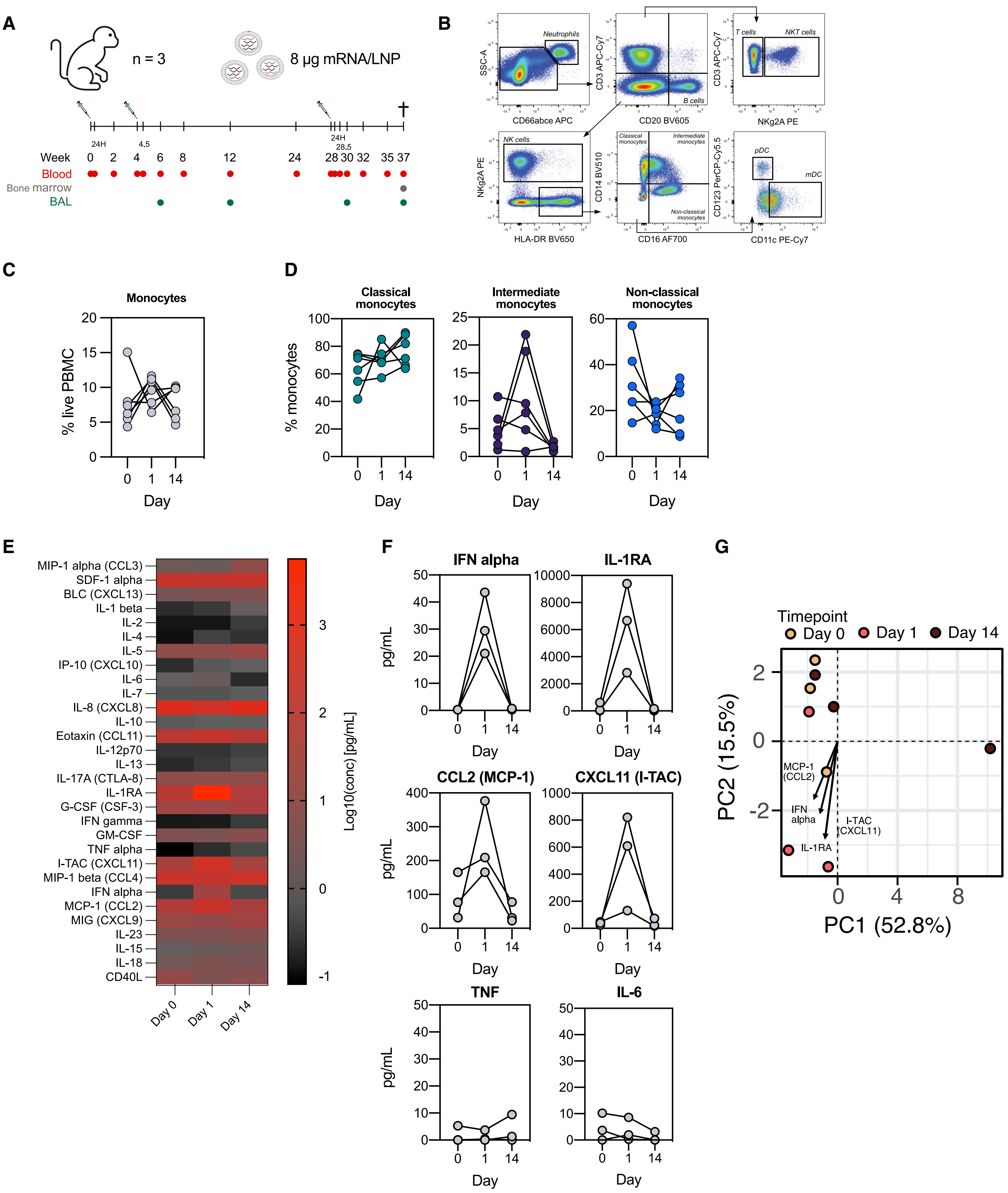

(legend on next page)

NHPs.[6–8] However, in NHP challenge studies, partial protection against SARS-CoV-2 was achieved, indicating that protective immunity had been established. The NHP model therefore offers an opportunity to perform high-resolution analyses in a physiologically relevant setting.

RESULTS

CVnCoV administration rapidly induced type I interferon-polarized and transient innate immune activation

We measured multiple aspects of the immune response induced by CVnCoV, including immediate responses after administration as well as long-term adaptive responses. Three rhesus macaques were immunized with 8 μg CVnCoV encapsulated in LNPs at weeks 0 and 4, followed by a booster dose at week 28. Peripheral blood and bronchoalveolar lavage (BAL) samples were collected over the 37-week study period, and bone marrow aspirates were collected after euthanasia at study end (Figure 1A).

Within 24 h of immunization, markers of innate immune activation and toxicity showed no or only minor transient fluctuations that remained within the normal range of the clinical chemistry and complete blood count (CBC) (Figures S1A and S1B). Animals did not show any behavioral changes, increase in body temperature, or long-term weight differences. By combining CBC and phenotyping by flow cytometry (Figure 1B), we were able to detect a transient decrease in circulating lymphocytes, including T cells, B cells, natural killer (NK) cells, and NK T cells 24 h after immunization (Figure S1C), coinciding with an elevated proportion of circulating monocytes (Figure 1C). This increase was mainly represented by $CD14^+$ $CD16^+$ intermediate monocytes (Figure 1D), which is consistent with previous reports on intermediate monocyte expansion after administration of TLR7/8-based adjuvants or mRNA vaccines.[9–11] Most of the 30 plasma analytes measured showed no or very low increases 24 h after CVnCoV administration (Figure 1E). However, in line with the transient increase in intermediate monocytes, elevated levels of monocyte attractant protein 1 (MCP-1; CCL2) were detected at 24 h (Figures 1E and 1F). Interleukin-1 receptor antagonist (IL-1RA) was induced as well as cytokines associated with a type I interferon (IFN) response, such as IFN-α and CXCL11 (Figures 1E and 1F). All cytokines had returned to baseline levels by day 14 (Figures 1E and 1F). No detectable levels of classic inflammatory cytokines, such as tumor necrosis factor alpha (TNF-α) and IL-6, were induced. Principal component analysis (PCA) of the 30 plasma analytes confirmed differences between baseline and 24-h samples, mainly because of the type I IFN-associated cytokines MCP-1 and IL-1RA (Figure 1G). This demonstrated that systemic innate immune activation was induced by CVnCoV with limited and transient adverse events.

A third dose increased the levels of neutralizing and cross-reactive antibodies

Using the 8-μg dose of CVnCoV, we found low but detectable antibody titers against SARS-CoV-2 S protein and the receptor-binding domain (RBD) of the spike (S) protein at week 6, 2 weeks after the second dose (Figure 2A). In clinical studies, suboptimal efficacy elicited by immunization with two doses of CVnCoV ultimately led to a halt in clinical development. We therefore investigated the potential of a third dose to increase the responses. The animals received a third dose 24 weeks (6 months) after the second dose, a relevant time frame for a human booster dose. Titers increased significantly after the third (booster) dose, which is in line with the kinetics of antibody responses in the clinical trial testing the booster potential of the third CVnCoV immunization.[12] Two weeks after the third dose, binding titers for the S protein and RBD were 12.8- and 6.4-fold higher, respectively, than peak titers 2 weeks after the second dose (Figure 2A). The boosting effect was even stronger for neutralizing (Figure 2B) and pseudovirus neutralizing titers (Figure 2C). These titers increased by 33.6- and 23.7-fold, respectively, and matched the neutralization capacity of the World Health Organization (WHO) international standard (NIBSC 20/136), which was not the case after the first two immunizations (Figure 2B). This large change in response from the second to the third dose for neutralization of the ancestral (WA-1) SARS-CoV-2 virus has not been reported for the licensed mRNA vaccines mRNA-1273 and BNT162b2, although titers were higher after the first two doses.[13–16] Although low-dose CVnCoV can elicit high responses with three immunizations, mean responses after three doses were still lower than those reported with the 30- to 100-μg doses of licensed mRNA vaccines in NHP models[17,18] or clinical studies,[19,20] although differences between the assays used have to be considered.

The significant increase in titers after the third CVnCoV dose was also reflected by higher neutralization potency, defined as the ratio between the neutralizing and the binding titers (Figure 2D).[13] This suggests not only a substantial improvement in titers but also in antibody quality; however, because the group size was small, and one animal persistently showed lower responses, this difference was not significant.

In a recent study, S protein was detected in the serum of BNT162b2 vaccinees after the first but not the second dose because of masking of S epitopes by the serum antibodies.[16] In our NHP sera, S protein was detectable 24 h after the first dose in all three animals, whereas only the animal with the lowest S-specific titers had systemically detectable S protein 24 h after the third dose (Figure 2E).

In addition to the large increase in antibody response to the ancestral S protein after the third dose, antibody titers against S proteins from

Figure 1. Innate immune response after mRNA immunization

(A) Study design. (B) Gating strategy used in immunophenotyping. (C and D) Total monocyte and monocyte subset frequency in blood after immunization. (E) Plasma cytokines after immunization, assessed by 30-plex Luminex assay. (F) Selected plasma cytokines after prime immunization (related to type I IFN response, TNF and IL-6 as controls). (G) PCA of plasma cytokines (30-plex assay). (C) and (D) combine data from 3 NHPs after the first and third immunizations. (E)–(G) focus on the innate immune response after prime immunization.

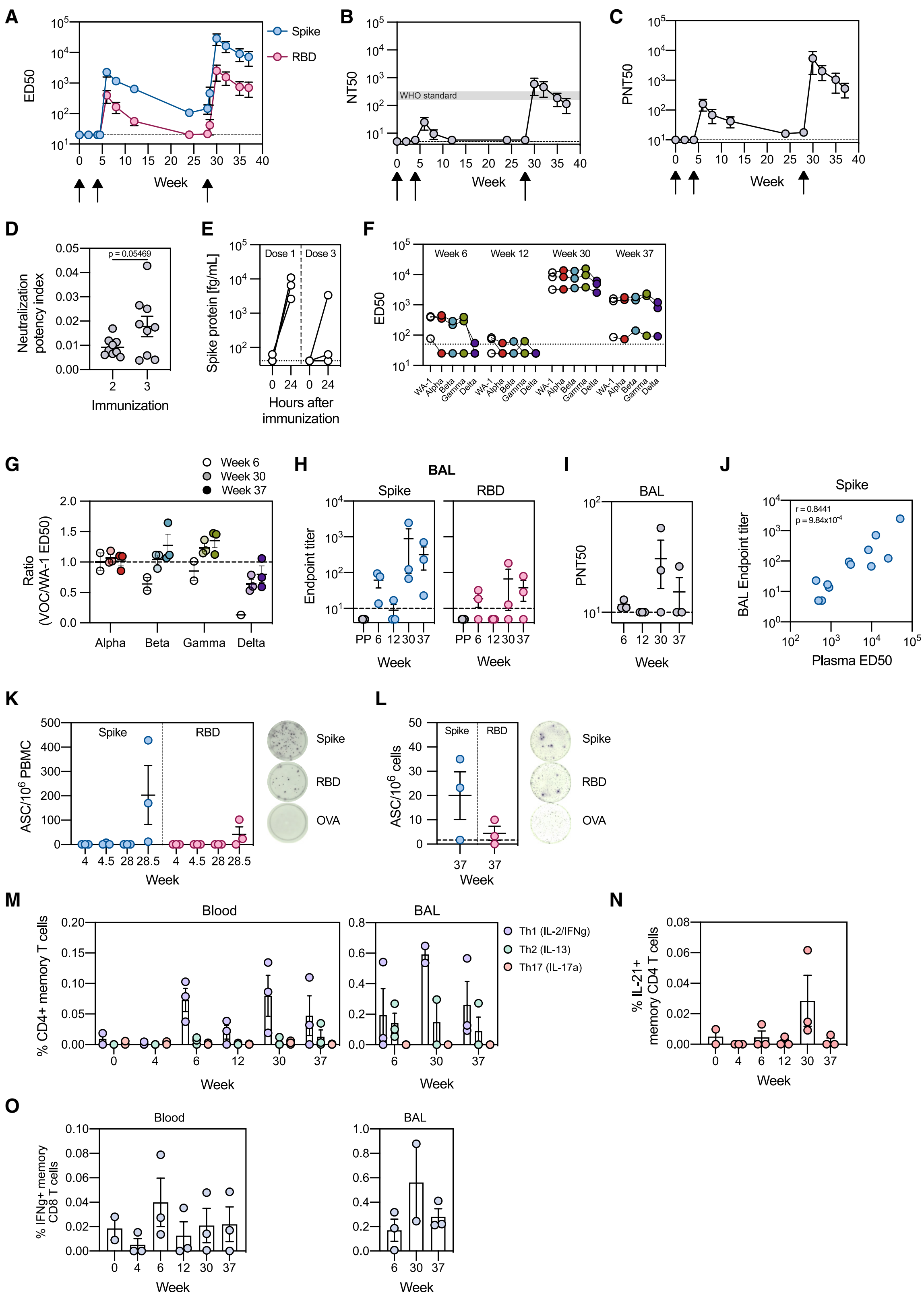

(legend on next page)

the SARS-CoV-2 B.1.1.7 (Alpha), B.1.351 (Beta), P.1 (Gamma), and B.1.617.2 (Delta) variants were also increased (Figure 2F). The increase after the third dose was also demonstrated by higher ratios between variant and ancestral binding titers (Figure 2G).

Although serum antibodies are often used to assess vaccine responses and predict correlates of protection,[21–23] it is likely that mucosal immunity to SARS-CoV-2 is necessary to prevent infection and mild disease. Anti-S and RBD-binding immunoglobulin G (IgG) and neutralizing titers in BAL fluid were barely detectable 2 weeks after the second dose, but, as with serum antibodies, they were strongly boosted after the third dose (Figures 2H and 2I). There was a strong correlation between antibody levels in BAL and in plasma ($r = 0.8441$, $p < 0.001$) (Figure 2J), suggesting that mucosal antibodies may predominantly transudate from serum, as proposed previously.[21]

Antibody-secreting S and RBD-specific plasmablasts were undetectable after the two priming immunizations but detectable by enzyme-linked immunospot (ELISpot) 4 days after the third dose (Figure 2K). S- and RBD-specific plasma cells in the bone marrow were found at week 37 (study end; Figure 2L), suggesting that the animals had generated vaccine-specific B cell populations critical for establishing longevity of the antibody response. S-specific memory T cells, assessed by an antigen recall assay using stimulation with overlapping S peptides and intracellular cytokine production (Figures S2A and S2B), showed low but detectable $CD4^+$ T cell responses after the second dose in the blood and BAL (Figure 2M). These responses waned but were boosted by the third dose, especially in the BAL. The T cell response was Th1 polarized, as shown by IFN-γ and IL-2 production, but a proportion of IL-21-producing circulating T follicular cells was also detected, especially 2 weeks after the third dose (Figure 2N). Low frequencies of IFN-γ producing $CD8^+$ T cell responses were detectable in the blood and BAL (Figure 2O).

A third dose 24 weeks after the two-dose primary series amplified the initial vaccine responses. A strong increase in antibody titers, resulting in improved neutralization of and binding to the ancestral strain as well as variant strains and higher mucosal responses, was accompanied by induction of S-specific, IL-21-secreting, circulating T follicular helper and Th1 memory cells.

The third immunization drives affinity maturation of vaccine-specific B cells

S-specific circulating memory B cells, measured by binding to fluorescently labeled S using flow cytometry (Figures 3A and S3A), showed a clear increase in frequency after the second immunization. Despite the expected waning, S-specific memory B cells remained at detectable levels for 24 weeks (Figure 3B). The third immunization resulted in a clear expansion with readily detectable levels until study end. Of the S-specific memory B cells, only 11.1% (range, 0%–30.3%) were specific to the RBD throughout the study, as reported previously (Figure 3C).[24–26] Conversely, on average, 64.9% (range, 33%–79%) of the antibodies in plasma were RBD reactive (Figures 3D, S3B, and S3C), similar to the proportions observed in convalescent individuals.[27] This discrepancy in the memory B cell pool and circulating antibodies may reflect differences between the antibody-producing plasma cells in the bone marrow compartment and memory B cells. Over time and with the third immunization, we observed that, although the proportion of RBD-specific antibodies decreased slightly, they still represented the majority of the response (Figure 3D).

To determine whether there were qualitative differences in affinity maturation and epitope specificities in vaccine-elicited B cell responses, we single-cell-sorted S-specific memory B cells obtained 2 weeks after the second and third immunizations and sequenced the variable regions of the heavy (VH) and light (VL) chains of their B cell receptors. Productive, high-quality sequences were obtained from a total of 444 single memory B cells (155 and 289 after the second and third immunizations, respectively). The level of somatic hypermutation (SHM) in the VH region was calculated after alignment with the largest germline IGHV allele database available, based on multiple rhesus macaques.[28] Significantly higher SHM was found in the memory B cells after the third immunization compared with the second (Figure 3E). Along with the increase in SHM after the third dose, we found that antibody binding avidity increased significantly at week 30, in agreement with a recent report.[29] High avidity remained stable until study end, another indication of a qualitative improvement of the humoral response (Figure 3F).

The memory B cell response was highly polyclonal, with the majority of the sequences belonging to independent lineages (defined as the same IGHV and IGHJ allele, same HCDR3 length, 80% amino acid identity in the HCDR3 and one identical HCDR3 junction) (Figure S3D). However, several lineages were detected at weeks 6 and 30 (labeled in the

Figure 2. Enhanced adaptive immune responses after the third dose

(A) Plasma binding antibody response to the ancestral S protein (S-2P) and RBD by ELISA. ED50, half-maximal effective dilution. (B) Live virus neutralization (NT_{50}) in serum. The gray shaded area represents the neutralization titer of the WHO international standard (NIBSC 20/136). NT50, half-maximal neutralizing titer. (C) VSV-based pseudovirus neutralization (PNT_{50}) in serum. PNT50, half-maximal pseudoneutralizing titer. (D) Antibody neutralization potency index representing the ratio between neutralizing (NT_{50}) and binding (ED_{50}) antibody titers in plasma. Data points after the second (weeks 6, 8, and 12) and third immunizations (weeks 30, 32, and 35) are shown, respectively. (E) S protein concentration in serum after the first and third immunizations. (F and G) Plasma antibody binding to variant S proteins (F) and ratio between variant and ancestral binding titers (G). (H and I) Ancestral S protein and RBD binding (H) and neutralizing antibodies (I) in BAL. PP, pre-pandemic BAL samples. (J) Correlation of plasma and BAL anti-S-protein antibody titers. (K and L) Vaccine-specific plasmablasts in blood (K) and vaccine-specific plasma cells in bone marrow (L), assessed using ELISpot. Representative wells are shown on the right. Data are background subtracted based on OVA wells. ASC, antibody-secreting cell. (M–O) Frequencies of S-protein-specific CD4 T helper cell subsets (M), circulating T follicular cells (N), and CD8 T cells (O) in blood and BAL at selected time points. All data are background subtracted based on the DMSO-only condition. Significance was assessed by Wilcoxon signed-rank test or Spearman correlation. Arrows indicate immunizations. Dotted lines indicate the limit of detection (LOD) of each assay, except in (F), where it represents the ratio of 1 (equal binding to ancestral and variant S protein). Data are represented as mean ± SEM.

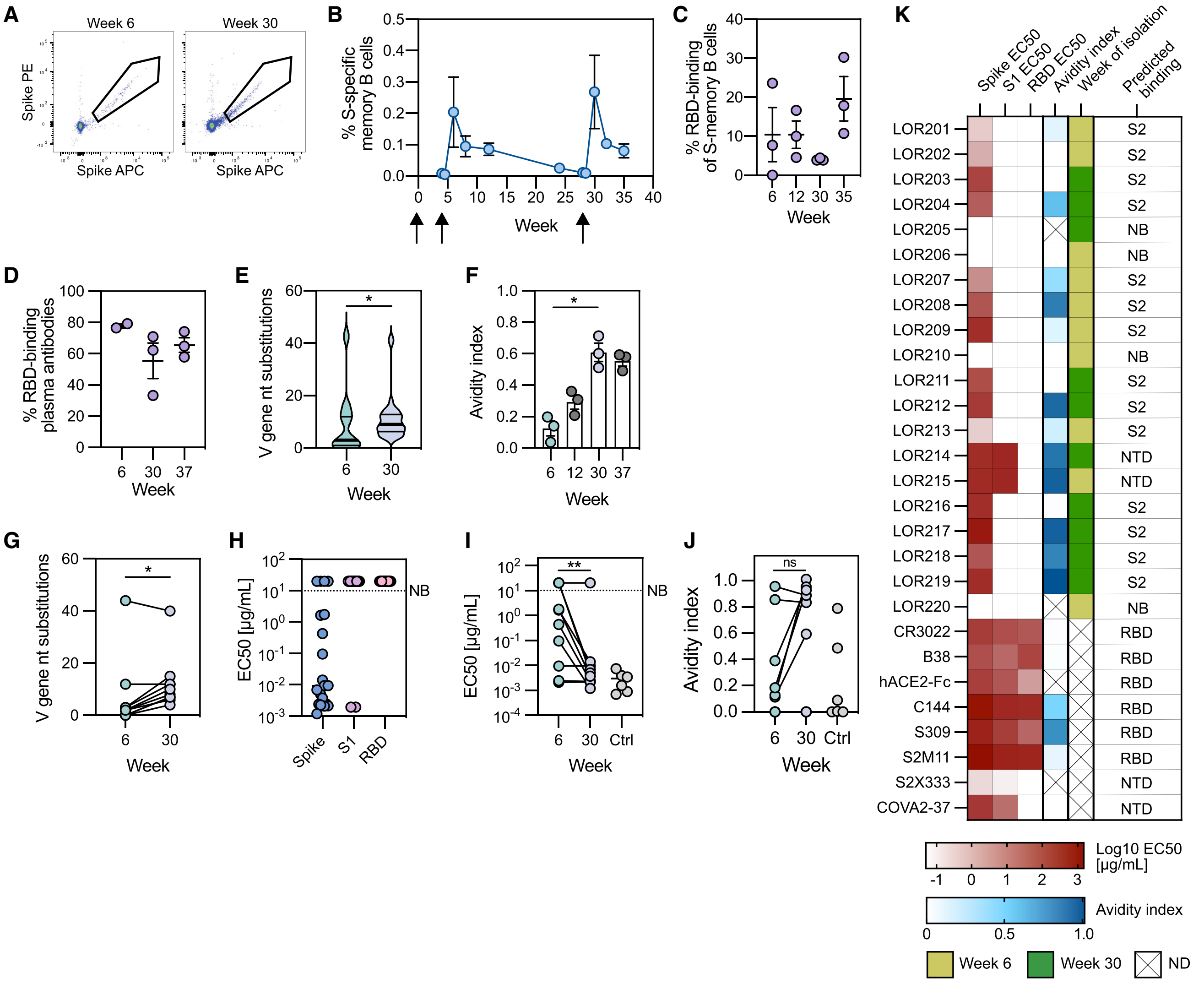

Figure 3. Maturation of the B cell response after the third immunization

(A) Representative gates used for sorting S-protein-specific IgG+ memory B cells 2 weeks after the second and third immunizations (weeks 6 and 30). (B) Longitudinal assessment of S-protein-specific memory B cells among IgG+ B cells. (C) Proportion of RBD-specific memory B cells among S-protein-specific memory B cells at selected time points. (D) Proportion of RBD-binding antibodies in serum at selected time points. (E) SHM in sorted memory B cells, presented as number of nucleotide substitutions in the heavy-chain V gene segment. (F) Avidity index of a polyclonal plasma antibody response. (G) SHM of expressed mAbs by time point of isolation. (H) Binding of expressed mAbs to ancestral S, the S1 subunit, and RBD. NB, non-binding. (I and J) mAb binding to ancestral S protein (I) and avidity index (J), plotted by time point of isolation, with previously characterized mAbs (Controls (Ctrl): CR3022, B38, hACE2-Fc, C144, S309, S2M11, S2X333, and COVA2-37) for comparison. (K) Heatmap depicting binding and avidity information with predicted subunit binding for isolated and control mAbs. ND, not determined. Statistical analysis was performed using non-parametric Mann-Whitney test (E) or Wilcoxon matched-pairs signed-rank test (F, G, I, and J). *$p < 0.05$, **$p < 0.01$. Data are represented as mean ± SEM.

same color in Figure S3D), indicating that they were maintained and expanded by the third dose. To investigate maturation of the B cell response after the third dose, we selected 10 sequence pairs from the lineages that were detected at weeks 6 and 30 and expressed them as monoclonal antibodies (mAbs). These selected lineages did not expand within the sampled B cell repertoire with the third dose (Figures S3E and S3F), but they showed significant affinity maturation (Figure 3G). This confirms the increased SHM as found in the memory B cell pool at large.

Although 16 of the 20 mAbs we expressed bound S protein, only two (from the same lineage) bound the S1 domain, and none of them bound RBD alone (Figure 3H). Therefore, we predict that most expressed mAbs bind the S2 subunit (Figure 3K), which is rarely a target of neutralizing antibodies. This corroborates our data on expansion of non-RBD-specific memory B cells with the booster immunization. High proportions of S2-specific B cells have been reported previously after SARS-CoV-2 infection[30] and vaccination.[31] Expressed mAbs

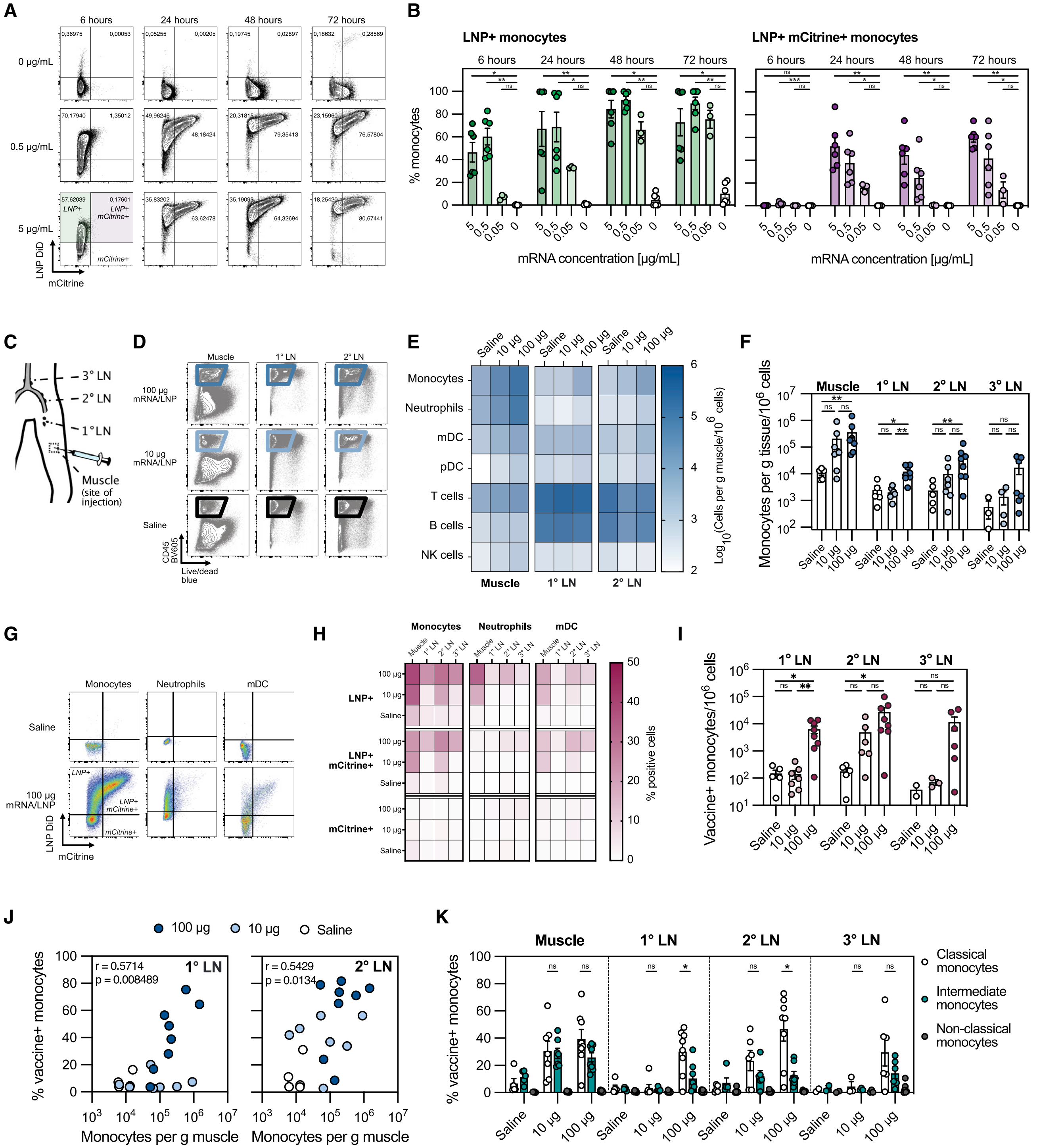

Figure 4. Tracking mRNA vaccines *in vivo* in NHPs

(A and B) *In vitro* uptake and translation of 0, 0.5, and 5 ug/mL mCitrine mRNA/LNPs in enriched primary human monocytes at 6, 24, 48, and 72 h. Shown are representative flow cytometry plots (A) and summarized data (B). (C) NHPs were immunized at 0 h with saline, 10 or 100 µg mRNA/LNP. They were sacrificed 24 h later, and draining as well as non-draining tissues were collected and analyzed. (D) Representative flow cytometry plots of $CD45^+$ immune cell infiltration in different tissues. (E) Enumeration of infiltrating cell subsets in different tissues. (F) Monocyte infiltration into the site of injection and draining LNs. (G) Flow cytometry plots of LNP DiD and mCitrine signal in draining LNs by innate cell subsets from saline- and vaccine-injected sites. (H) Proportion of vaccine-positive cells in different tissues by the innate cell subset. (I) Vaccine-positive

(legend continued on next page)

isolated from B cells at week 30 showed significantly better binding to S protein than the related sequences isolated at week 6 (Figure 3I). The mAbs from week 30 exhibited a trend toward higher-avidity indices, which were comparable with well-characterized reference mAbs specific for the N-terminal domain (NTD) or RBD of the ancestral S protein (Figure 3I). This shows a clear maturation of the B cell response with the third vaccine dose, demonstrated at the antibody and memory B cell level.

A higher mRNA vaccine dose increases dissemination to more lymph nodes

We and others have shown in NHPs that mRNA vaccine administration leads to local inflammation in the muscle at the site of injection, consisting of infiltration of immune cells, including antigen-presenting cells; uptake; and translation of the mRNA.[32,33] The efficiency of this process is probably affected by the dose of the vaccine, so the low dose used in our study may have limitations regarding the number of cells infiltrating the injection site and becoming available as target cells for the vaccine as well as for disseminating vaccine antigen. This would have consequences for initiation of an adequate vaccine-specific response. We used an mRNA construct based on sequence-optimized unmodified mRNA encoding the fluorescent protein mCitrine that enabled identification of mRNA translation in cells. The mRNA construct was formulated in DiD-labeled LNPs, allowing us to track LNP uptake independent of mRNA translation. By exposing isolated monocytes to 5 μg/mL mRNA/LNP *in vitro*, we found detectable LNP uptake already after 6-h incubation (Figures 4A and 4B). mCitrine expression was slightly delayed compared with LNP uptake but was detectable at 24 h, and high levels of mCitrine$^+$ cells remained detectable for at least 3 days. Using a 10-fold higher dose of the construct did not result in higher LNP uptake, but we did observe a dose-dependent pattern of mCitrine expression *in vitro*.

We then investigated whether differences in doses significantly affected biodistribution and antigen expression *in vivo*, mimicking low-dose CVnCoV administration. Rhesus macaques received intramuscular injections of low (10 μg) or high (100 μg) doses of the labeled LNP/mCitrine mRNA construct (Figure 4C) with intramuscular saline injections as controls. The animals were injected at four different sites simultaneously (left and right deltoid and quadricep muscles), allowing direct comparison of saline versus mRNA vaccine administration in the same animal. This enabled collection of multiple data points from each animal while limiting the number of animals used. Injection site muscle biopsies were taken after 24 h because we have observed previously that there is a high level of antigen uptake and local innate immune activity at this time point.[32,34,35] To identify which lymph nodes (LNs) were primarily targeted by mRNA vaccination, several LN clusters were collected and classified as primary (1°; axillary or inguinal), secondary (2°; apical or iliac), or tertiary/third (3°; supraclavicular or para-aortic) draining LNs based on their proximity to the injection site (Figure 4C). We analyzed early immune processes critical for initiation of adaptive responses, such as cell infiltration, vaccine uptake and translation, and dissemination to LNs.

Compared with the control tissues, there was noticeable recruitment of CD45$^+$ immune cells to the LNP/mRNA injection sites as well as into the draining LNs specifically in a dose-dependent manner (Figure 4D). Multiple cell subsets were defined within CD45$^+$ immune cells (Figure S4A). CD66abce$^+$ neutrophils, classic CD14$^+$ CD16$^-$ monocytes, and myeloid dendritic cells (mDCs) were the most frequent cell types infiltrating the muscle injection site (Figure 4E), with some infiltration of the muscle by plasmacytoid DCs, T cells, B cells, and NK cells. Of these, monocytes exhibited the most pronounced dose-dependent accumulation in the draining LNs after mRNA vaccine administration (Figures 4E and 4F).

mDCs and monocytes play an essential role in antigen presentation and maintaining adaptive responses. Using mRNA encoding for mCitrine, we determined the target immune cells for the LNP/mRNA vaccine (Figure 4G). No LNP$^+$ or mCitrine$^+$ cells were detected at the saline-injected control sites, demonstrating that uptake and translation are restricted to the vaccination sites and their draining LNs (Figures 4G and 4H). Although we observed that non-immune CD45$^-$ cells in muscle tissue were able to take up LNP and translate mRNA into protein (Figure S4B), production of mCitrine was much less efficient compared with CD45$^+$ immune cells. Monocytes were found to be the most abundant LNP$^+$ mCitrine$^+$ immune cells (Figure 4H), with classic CD14$^+$ CD16$^-$ and intermediate CD14$^+$ CD16$^+$ subsets infiltrating the site of injection, although classic monocytes were the predominant vaccine$^+$ subset in the draining LNs (Figure 4K). mDCs also showed clear LNP uptake and mCitrine translation (Figures 4G and 4H). We observed that a 10-fold higher dose of vaccine led to broader mRNA dissemination, evidenced by LNP$^+$ mCitrine$^+$ monocytes in more draining LN clusters (Figure 4I).

In contrast to the clear expression of mCitrine in monocytes and mDCs, neutrophils were efficient at internalizing LNP but not at translating the mRNA (Figures 4G and 4H), in accordance with our earlier data.[33] Other immune cells, such as T cells, B cells, and plasmacytoid DCs, showed low signals for LNP and mCitrine in comparison with monocytes and mDCs (Figure S4C). There was a correlation between the number of infiltrating monocytes in the muscle and the number of mCitrine$^+$ monocytes in 1° and 2° draining LNs (Figure 4J). This demonstrates that a sequence-optimized unmodified mRNA vaccine has a similar pattern of biodistribution and cell-specific targeting as that reported for modified mRNA vaccines, but a low dose of mRNA results in restricted dissemination to the 2° lymphoid organs compared with a higher dose.

monocytes in 1°, 2°, and 3° draining LNs 24 h after immunization. (J) Correlation between the number of infiltrating monocytes in the muscle and the number of vaccine-positive monocytes in 1° and 2° draining LNs. (K) Vaccine signal in different monocyte subsets and tissues at 24 h. Statistical analyses were performed using a non-parametric Kruskal-Wallis test or Spearman correlation. *$p < 0.05$, **$p < 0.01$. ns, not significant. Data are represented as mean ± SEM.

DISCUSSION

The 48.7% efficacy against COVID-19 of any severity observed in the phase IIb/III clinical trial of CVnCoV was followed by the decision to reorient the development of this vaccine candidate. However, the results from this NHP study showed that a third CVnCoV dose significantly enhanced the magnitude and quality of the immune response compared with two doses. Although the average neutralizing antibody responses after three 8-μg doses of CVnCoV were numerically lower than those reported with the 30 to 100-μg doses of the licensed mRNA vaccines in NHPs[17,18] or humans,[19,20] the marked fold increase in neutralizing activity against ancestral SARS-CoV-2 after the third dose, compared with after the second dose, was substantially higher than that reported for three doses of the licensed mRNA vaccines mRNA-1273 and BNT162b2.[13–15] Establishment of vaccine-specific plasma cells in the bone marrow after the third dose, combined with neutralizing serum antibody titers in the range of the WHO international standard 2.5 months after the last dose suggests that three CVnCoV doses elicit durable immune responses beyond the investigated study period. Similar to immunization with BNT162b2,[16] we detected S protein in sera of CVnCoV-vaccinated NHPs 24 h after administration but at a concentration 10-fold lower than that observed in BNT162b2 vaccinees. This may reflect the overall antigen load and affect development of the vaccine responses, although the physiological differences in size and weight between humans and NHPs need to be considered. Our study results suggest that a third CVnCoV dose provides efficient boosting of the immune responses when SARS-CoV-2-specific memory has been established, and it provides mechanistic information about how this boosting effect is brought about. In recent clinical studies, CVnCoV performed well as a booster vaccine in previously vaccinated individuals,[12,36] eliciting superior antibody titers compared with Valneva's alum/CpG-adjuvanted inactivated vaccine candidate VLA2001 but inferior responses to licensed mRNA vaccines.[36] We found clear evidence of qualitative enhancement of the responses in plasma antibodies and the B cell repertoire, manifested by higher binding avidity and SHM, respectively. We also showed that the third immunization enhanced cross-reactivity of plasma antibodies to several variant S proteins, which is relevant in the light of continuing emergence of new variants with divergent mutations in the S protein.[37]

The suboptimal responses to CVnCoV in clinical trials spurred development of the next-generation vaccine candidate CV2CoV. This updated version of CVnCoV contains unmodified nucleosides but with optimized non-coding regions and has been reported to induce higher titers of binding and neutralizing antibodies, memory B cell responses, and T cell responses as well as more robust protection compared with CVnCoV when two doses were administered 4 weeks apart in NHPs.[8] The S-specific antibody and B and T cell responses after the third dose of CVnCoV found in our study were in a range similar to that found for CV2CoV after two doses. Although our study did not include virus challenge, the serum neutralizing titers after the third dose were consistent with titers reported in other studies that provided significant protection in challenge experiments.[8,38] Based on these published data, protection from infection would presumably be higher after three doses of CVnCoV compared with two and in the range observed with CV2CoV. However, CV2CoV also induced higher levels of type I IFN responses and MIP-1α 24 h after immunization compared with CVnCoV. This needs to be evaluated further because undesired side effects as a result of innate immune activation have been a concern with unmodified mRNA vaccines.

The first unmodified mRNA vaccine tested in humans was against rabies virus; about 78% of study participants reported transient mild to moderate systemic side effects after administration of protamine-complexed mRNA.[39] More recently, in a phase 1 clinical trial, considerable side effects were reported with 5 μg unmodified rabies mRNA in LNPs, whereas 1- or 2-μg doses were well tolerated and elicited immune responses comparable with those of a licensed rabies vaccine.[40] These data on dosing contributed to the design of the doses selected for CVnCoV, which also displayed dose-dependent increases in reactogenicity up to the maximum dose of 12 μg tested.[5]

The innate immune activation characterized by type I IFN responses after mRNA vaccination likely plays an important role in the immunogenicity of the mRNA platform and its Th1 polarized profile of adaptive responses.[34,41,42] Type I IFN responses have been shown to directly support B cell differentiation and survival, resulting in enhanced antibody responses.[43,44] Increased antibody half-life and durability of humoral responses have been shown with type I IFN-inducing adjuvants, such as TLR3, TLR7/8, and TLR9 ligands (poly(IC:LC), R848, CpG).[45–48] In the current study, we observed that CVnCoV induced a strong, transient type I IFN response (IFNα, CXCL11) as well as monocyte activation, evidenced by MCP-1 induction and intermediate monocyte differentiation within 24 h of administration. A similar activation profile after administration of nucleoside-modified mRNA vaccines has been reported previously.[11,32,49] Intermediate monocytes have been shown to be important for antigen presentation to $CD4^+$ T cells[50] and support differentiation of naive B cells into antibody-secreting plasmablasts.[51,52]

Little is known about the mechanisms of action by which LNP/mRNA-based vaccines generate strong vaccine responses. Using an *in vitro* experimental approach, we showed that lower doses of mRNA led to detectable protein production in fewer monocytes compared with higher doses. Antigen availability is an important determinant of the outcome of the germinal center reaction,[53] and a low protein translation rate could result in less protein antigen being available to support the germinal center reaction, limiting the B cell response. Another major finding of our study is that sequence-optimized unmodified and modified mRNA/LNP vaccine formulations appear to have a similar biodistribution pattern and cell-specific targeting. Our *in vivo* biodistribution data showed that a lower dose resulted in a limited spread of vaccine-positive immune cells and fewer targeted LNs, demonstrating restricted vaccine dissemination. This restricted dissemination would have a greater effect in a primary immunization setting, where induction of vaccine-specific immune responses relies on encounters between the vaccine and sparse

antigen-specific naive lymphocytes, than in a booster setting, when memory B and T cell pools would already have been established.

Although this study has obvious limitations because of having only three animals per group, we followed the animals with multiple samplings over 8 months, and we were able to analyze numerous aspects of the immune responses. Therefore, we were able to study the evolution of the vaccine responses over a significant time period with control measurements from the same individual. We mapped antibody responses in detail and monitored the emergence of S-specific memory B cells over time. After two doses, most serum antibodies were RBD-specific. The third immunization had the particular effect of expanding the immune response against non-RBD epitopes, proportions of which remained stable until study end. This is important because SARS-CoV-2 vaccination has been shown to induce responses that are dominated by non-neutralizing antibodies,[54,55] and although animal studies have shown that non-neutralizing antibodies can contribute to protection,[56,57] clinical studies have shown that serum neutralizing antibody titer strongly correlates with vaccine-induced efficacy against symptomatic COVID-19.[23,58] In this study, NHPs developed high titers of neutralizing antibody after the third dose. However, none of the mAbs we expressed from the S-specific circulating memory B cell pool were RBD reactive. This probably reflects the high representation of non-RBD-specific clones in the B cell repertoire, as reported elsewhere,[31,55] and warrants further investigation of immunization strategies to expand the RBD-specific B cell repertoire.

Our study in NHPs adds important mechanistic information on CVnCoV, including use of a three-dose immunization regimen that has been reported in a clinical trial.[12,59] Our data will complement those observed in human volunteers to elucidate the mechanism of action of CVnCoV and inform development of an improved version for future use.

MATERIALS AND METHODS

Vaccines

The CVnCoV vaccine candidate is based on the RNActive platform. It has a 5′ cap structure, 5′ UTR, a GC-enriched open reading frame, 3′ UTR, and poly(A) tail and no chemically modified nucleosides. The mRNA was encapsulated using the LNP technology of Acuitas Therapeutics (Vancouver, BC, Canada). The LNPs used in this study are particles of ionizable amino lipid, phospholipid, cholesterol, and a PEGylated lipid. The mRNA-encoded protein is based on the S-protein of the SARS-CoV-2 NCBI reference sequence (NCBI: NC_045512.2, GenBank: YP_009724390.1) and encodes for the full-length S-protein featuring K986P and V987P mutations. The mRNA-encoded protein for mCitrine is based on the description by Griesbeck et al.[60]

Immunogenicity study design and sample collection

Three female Chinese rhesus macaques (*Macaca mulatta*, 12–13 years old) were used in this study. They were housed in the Astrid Fagraeus Laboratory at Karolinska Institutet (Stockholm, Sweden). All animal experiments were conducted following the guidelines and regulations of the Association for Assessment and Accreditation of Laboratory Animal Care and the Swedish Animal Welfare Agency. The study was approved by the regional animal ethics committee of Northern Stockholm. The animals received intramuscular (i.m.) injections of 8-μg doses of CVnCoV in their left quadriceps at weeks 0, 4, and 28. Heparinized peripheral blood, serum, and BAL samples were collected over the 37-week study period, and bone marrow aspirates were collected after euthanasia at the end of this period. Body weight and temperature were monitored at each sampling time point. CBC and clinical chemistry analyses were performed at baseline and 24 h and 14 days after the first and third doses by Adlego Biomedical (Solna, Sweden). Clinical chemistry was performed on an Abaxis Vetscan VS2 3.1.35 chemistry analyzer using mammalian liver profile rotors (Triolab, Solna, Sweden).

Sample processing

Peripheral blood mononuclear cells (PBMCs) were isolated by standard gradient density centrifugation from heparinized blood using Ficoll-Paque (GE Healthcare). PBMCs were used immediately for downstream applications or cryopreserved in 10% dimethyl sulfoxide (DMSO)/fetal calf serum (FCS) until use.

BAL cells were separated from the supernatant by centrifugation. Cells were passed through a 70-μm cell strainer and used fresh in a T cell stimulation assay. Supernatants were stored separately and concentrated 10-fold using Amicon-Ultra centrifugal filter units with 30-kDa cutoff (Millipore) before downstream analysis.

Innate response flow cytometry

Immune cell subsets in peripheral blood were monitored by flow cytometry on days 0, 1 and 14 after the first and third doses. Freshly isolated PBMCs were stained with Live/Dead Fixable Blue Dye (Life Technologies) and FcR blocking reagent (Miltenyi Biotec) followed by a panel of antibodies for innate immunophenotyping (Table S1), washed, and fixed in 1% paraformaldehyde (PFA). Samples were acquired on a BD LSRFortessa cell analyzer, and the data were analyzed using FlowJo software v.10.7.1 (FlowJo).

Plasma cytokine and chemokine quantification by Luminex

Plasma cytokine and chemokine analyses at baseline and 24 h and 14 days after the first dose were performed using the ProcartaPlex NHP Cytokine & Chemokine Panel 30plex (Thermo Fisher Scientific) according to the manufacturer's instructions. Samples were analyzed using a MagPix (Luminex) instrument, and the data were analyzed with Belysa Immunoassay Curve Fitting software (Millipore). Standard curves were generated using 5-parameter logistic (5-PL) curve fit.

NHP plasma ELISAs

Recombinant proteins were acquired through the Global Health-Vaccine Accelerator Platform (GH-VAP) funded by the Bill & Melinda Gates Foundation (Seattle, WA, USA). Polyclonal antibody

responses elicited in blood and BAL were analyzed using 96-well half-area ELISA plates coated with recombinant antigens (SARS-CoV-2 prefusion [S-2P] stabilized S protein, RBD, and variant S proteins [HexaPro S backbone]) in PBS at 1 μg/mL overnight at 4°C. Plates were washed three times with PBS containing 0.05% Tween 20 (PBS-T) and blocked with blocking buffer (PBS + 5% skim milk powder) for 1 h at room temperature (RT). Samples were serially diluted in blocking buffer, added to the ELISA plate, and incubated for 2 h at RT. For the RBD competition ELISA, samples were pre-incubated with or without 20 μg/mL RBD in blocking buffer for 30 min before being added to S-coated ELISA plates. For the chaotropic wash ELISA, plates were treated with 1.5 M NaSCN or PBS for 10 min at RT after sample incubation. Plates were washed three times, and goat anti-monkey IgG-horseradish peroxidase (HRP) (Nordic MUBio) in blocking buffer was added to the plate for 1 h at RT. Plates were developed using 1-Step Ultra TMB-ELISA substrate (Thermo Fisher Scientific), and the reaction was stopped with 1 M H_2SO_4. The plates were read at 450 nm with background correction at 550 nn. The data were analyzed with Prism 9.2.0 using 4-parameter logistic (4-PL) curve fit. Proportions of RBD-binding antibodies were determined from a decrease in ED_{50} when RBD was added as a competitor. The avidity index was calculated from the ratio of ED_{50} values between PBS and NaSCN conditions.

mAb ELISAs

For mAb characterization, the ELISA was performed as above with the following modifications. Plates were coated with 4 μg/mL recombinant protein overnight at 4°C, washed, and blocked as described. Samples were serially diluted in blocking buffer and added to the ELISA plate. In the chaotropic wash ELISA, 1.0 M NaSCN or PBS was used to assess the strength of the binding interaction. After sample incubation for 2 h, plates were washed, and goat anti-human IgG-HRP, Fc specific (Jackson ImmunoResearch), in blocking buffer was added for 1-h incubation. Plate development and data analysis were performed as described above.

Recombinant hACE2-human Fc fusion protein and previously characterized S protein-specific mAbs were used as references: CR3022,[61] B38,[62] C144,[63] S309,[64] SM211,[65] S2X333,[66] and COVA2-37.[67]

Neutralization assays

The live virus neutralization assay was performed at Vismederi (Sienna, Italy) using SARS-CoV-2 2019-2019-nCoV strain 2019-nCov/Italy-INMI1 clinical isolate as described previously.[68] Briefly, serial 2-fold dilutions of heat-inactivated serum samples, starting at 1:10, were mixed with an equal volume of viral solution with 100 $TCID_{50}$ of SARS-CoV-2 and incubated for 1 h before being transferred in duplicate to plates containing semi-confluent Vero E6 monolayers. The plates were incubated for 4 days at 37°C and 5% CO_2. After 4 days, the plates were inspected using an inverted optical microscope. The highest serum dilution that protected more than 50% of cells from cytopathic effects was designated as the NT_{50}. The first WHO international standard for anti-SARS-CoV-2 Ig (NIBSC 20/136) was analyzed in parallel to NHP samples for comparison.

Pseudovirus particle neutralization assays

The pseudovirus particle neutralization assay (PNA) was performed at Nexelis (Laval, QC, Canada) using vesicular stomatitis virus (VSV)ΔG S pseudotyped virus with a luciferase reporter as described previously.[69] Briefly, serial 2-fold dilution series of heat-inactivated serum were incubated with a constant amount of pseudotyped virus particles and then transferred onto Vero E6 cells in 96-well plates. Test plates were incubated at 37°C and 5% CO_2 overnight. The next day, luciferase substrate was added to the plates, which were read using a luminescence microplate reader equipped with SoftMax Pro GxP software (v.6.5.1. or higher). The assay was run in duplicate, and the serum dilution that neutralizes 50% of the pseudovirus particles (PNT_{50}) was interpolated by linear regression of the two serum dilutions flanking 50% of the control luminescence signal.

Electrochemiluminescence-based S antigen detection

SARS-CoV-2 S antigen was quantified using the S-PLEX SARS-CoV-2 Spike Kit (K150ADJS, Meso Scale Diagnostics, MD, USA) according to the manufacturer's instructions at the SciLifeLab Affinity Proteomics Unit (Uppsala, Sweden). For the analysis, a 25-μL sample was used, and the plates were read using a MESO QuickPlex SQ 120 instrument. An 8-point calibration standard curve, based on the recombinant SARS-CoV-2 protein included in the kit, was used to convert raw signals into data expressed in femtograms per milliliter.

T cell stimulation

To assess the frequency of S-protein-specific memory T cells in the blood and BAL, T cell re-stimulation with overlapping peptides was performed as described previously.[10] Briefly, 1.5×10^6 PBMCs or BAL cells were cultured in 0.2 mL complete medium (RPMI 1640 medium supplemented with 10% heat-inactivated FCS, 100 U/mL penicillin, 100 μg/mL streptomycin, and 2 mM L-glutamine) in a 96-well plate at 37°C, 5% CO_2. The cells were stimulated with 2 μg/mL PepMix SARS-CoV-2 overlapping peptide library in DMSO (15mers with 11-amino-acid overlap, JPT Peptide Technologies), spanning the whole S protein, or an equal volume of DMSO only in the presence of 10 μg/mL brefeldin A (Life Technologies). After overnight stimulation, cells were stained with Live/Dead Fixable Blue Dye (Life Technologies), surface stained, fixed and permeabilized with the Cytofix/Cytoperm Kit (BD Biosciences), and stained intracellularly with the panel of antibodies listed in Table S2. Cells were washed after staining and fixed with 1% PFA. Samples were acquired on a BD LSRFortessa cell analyzer, and the data were analyzed using FlowJo software v.10.7.1 (FlowJo). DMSO-stimulated cells were used for background subtraction.

S-protein-specific memory B cell quantification and sorting

The recombinant SARS-CoV-2 prefusion stabilized S protein and RBD were biotinylated using an EZ-Link Micro Sulfo-NHS-LC Biotinylation Kit (Thermo Fisher Scientific) according to the manufacturer's instructions. Probes were generated by coupling biotinylated

proteins to fluorophore-conjugated streptavidin (SA) molecules for detection by flow cytometry (SA-BV421, SA-APC, and SA-PE, BioLegend). Isolated PBMCs were stained by a panel of antibodies listed in Table S3 as well as S protein and RBD probes (S-APC, S-PE, and RBD-BV421, 100 ng each). The samples were analyzed on a BD Aria III Fusion cell sorter (weeks 6 and 30) or a BD LSRFortessa flow cytometer (all other time points). At weeks 6 and 30, memory B cells ($CD3^-$ $CD11c^-$ $CD14^-$ $CD16^-$ $CD123^-$ HLA-DR^+ $CD20^+$ IgM^- IgG^+) double-positive for S protein binding were single cell sorted into 96-well plates and frozen immediately on dry ice for subsequent B cell receptor (BCR) amplification. Data were analyzed using FlowJo v.10.7.1 (FlowJo).

Single-cell VDJ amplification and Sanger sequencing

RNA extraction and reverse transcription using random hexamers from single-cell-sorted S protein-specific memory B cells were performed using the Superscript III reverse transcriptase (Invitrogen) according to the manufacturer's instructions. Nested PCR was performed using reagents and procedures as reported previously for heavy and light Ig chains.[70] PCR products were Sanger sequenced by Genewiz (Leipzig, Germany), the chomatograms were preprocessed using scifer (v 0.99.3, DOI: 10.18129/B9.bioc.scifer) and the resulting sequences were aligned to the Karolinska macaque database KIMDB (1.0)[28] using IgDiscover.[71]

Sequence data analysis and mAb selection

Clonally related sequences were identified using heavy chain sequences and the Clonotypes module of IgDiscover,[71,72] applying established criteria to define a clone: same V and J gene assignment, same CDRH3 length, 80% similarity in CDRH3 and one identical CDRH3 junction.[32] Several lineages were detected at week 6 and week 30, and the respective antibody sequences were selected for mAb expression and characterization. Cloning, expression, and purification of mAbs as human IgG1 in mammalian cells were performed by Genscript (Leiden, the Netherlands).

Antigen-specific antibody-secreting cell detection in blood

Antigen-specific plasmablasts were enumerated by ELISpot on the day of the booster dose and 4 days thereafter. Multiscreen IP filter ELISpot 96-well plates (Millipore) were activated with 35% ethanol for 1 min and washed three times with PBS. Plates were coated overnight with 1 μg/mL Affinity Pure goat anti-human IgG Fc fragment-specific antibody (Jackson ImmunoResearch). The next day, plates were washed three times with PBS and blocked with complete medium for 1.5 h. Serially diluted, freshly isolated PBMCs were incubated overnight at 37°C, 5% CO_2. After incubation, plates were washed six times with PBS-T (0.05%) and incubated with biotinylated probes for 1.5 h (0.25 μg/mL goat anti-human IgG Fc fragment-specific antibody [Jackson ImmunoResearch], 1 μg/mL prefusion-stabilized S-protein, 1 μg/mL RBD, or 1 μg/mL ovalbumin (OVA)) to detect total IgG and antigen-specific IgG-producing cells, respectively. The plates were washed six times with PBS-T and incubated with 1:1,000 diluted SA-conjugated alkaline phosphatase (Mabtech) for 30 min. After another round of washing, plates were developed with nitro blue tetrazolium 5-bromo-4-chloro-3′ indolyphosphate (BCIP/NBT) precipitating substrate (Mabtech) for 5 min. An AID ELISpot reader (Autoimmun Diagnostika) was used to obtain spot counts. Ovalbumin wells were used for background subtraction.

In vitro uptake and translation of mCitrine LNP/mRNA

Primary human monocytes were isolated from buffy coats using RosetteSep Human Monocyte Enrichment Cocktail (STEMCELL Technologies) and gradient density centrifugation using Ficoll-Paque (GE Healthcare) according to the manufacturer's instructions. After isolation, 1.5×10^6 monocytes were cultured in 0.5 mL complete medium (RPMI 1640 medium supplemented with 10% heat-inactivated FCS, 100 U/mL penicillin, 100 μg/mL streptomycin, and 2 mM L-glutamine) at 37°C and 5% CO_2 in the presence of mCitrine-encoding mRNA in DiD-labeled LNPs at different mRNA concentrations (0.0–5.0 μg/mL) for the indicated amount of time (6–72 h). After culture, cells were washed with PBS, stained with Live/Dead Fixable Blue Dye (Life Technologies) and FcR blocking reagent (Miltenyi Biotec), followed by surface staining with the panel of antibodies listed in Table S4. Cells were then washed with PBS and fixed with 1% PFA. Samples were acquired on a BD LSRFortessa cell analyzer, and the data were analyzed using FlowJo software v.10.7.1 (FlowJo).

Biodistribution immunizations and sample collection

To study the innate immune responses to different mRNA doses, rhesus macaques received four i.m. doses into marked injection sites, one in each limb. Vaccines (0.5 mL/injection) contained saline as control or 10 or 100 μg mCitrine-encoding mRNA in DiD-labeled LNP. Sites of injection as well as vaccine-draining and non-draining tissues were sampled at necropsy 24 h after immunization and stored in RPMI 1640 medium on ice as described previously.[35,73]

Biodistribution experiment sample processing

Muscle biopsies were dissected and weighed before digestion into single-cell suspensions as described previously.[35,73] Briefly, muscle tissue was incubated at 37°C for 2 h without agitation in the presence of 0.25 mg/mL Liberase TL (Roche) and 0.5 mg/mL DNase I (Sigma). Enzyme activity was quenched by addition of complete medium, the mixtures were filtered through 70-μm cell strainers twice, and single-cell suspensions were washed before proceeding to the next step. Liver and bone marrow samples were processed by standard gradient density centrifugation in the same manner as blood samples. Lymphoid tissues were mechanically disrupted using a plunger and a 70-μm cell strainer and washed with complete medium. When sample processing was complete, single-cell suspensions were immediately stained for flow cytometry analyses.

Biodistribution experiment flow cytometry

To quantify the mCitrine vaccine signal in different immune cell populations, cell suspensions corresponding to approximately 2 g of muscle tissue or 5 million LN cells were stained for flow cytometry. First, cells were stained with Live/Dead Fixable Blue Dye (Life Technologies) and FcR blocking reagent (Miltenyi Biotec),

followed by a panel of antibodies listed in Table S4. Samples were then washed and fixed with 1% PFA. Before acquisition, AccuCount beads (Spherotech) were added to each sample for quantification according to the manufacturer's instructions. Samples were acquired on a BD LSRFortessa flow cytometer cell analyzer, and the data were analyzed using FlowJo v.10.7.1 (FlowJo).

Statistics

No statistical methods were used to predetermine sample size. The results were considered statistically significant when $p < 0.05$. For comparison of two groups of paired and unpaired samples, non-parametric Wilcoxon matched-pairs signed-rank test and Mann-Whitney U test were used, respectively. For comparison of three of more groups, non-parametric Kruskal-Wallis test with Dunn's multiple comparison test was used. Correlations were assessed using non-parametric Spearman's correlation. Analyses were performed in GraphPad Prism 9.

Data availability statement

BCR sequencing data have been deposited to GenBank: OP572523–OP573208.

SUPPLEMENTAL INFORMATION

Supplemental information can be found online at https://doi.org/10.1016/j.omtm.2022.10.001.

ACKNOWLEDGMENTS

We thank the team from Affinity Proteomics-Stockholm at SciLifeLab Sweden for technical support and generation of systemic cytokine data for this project. We acknowledge Affinity Proteomics-Uppsala at SciLifeLab Sweden for providing assistance with protein analyses. We thank Lauren Carter and the Nanoparticle Core Laboratory at the University of Washington Institute for Protein Design for protein reagents and Holger Kanzler from the Bill & Melinda Gates Foundation for input and insight throughout the study. Keith Veitch (keithveitch communications, Amsterdam, the Netherlands) and Margaret Haugh (CureVac) provided editorial support for the manuscript. This work was supported by grants from the Bill & Melinda Gates Foundation (OPP1192908 and INV-017217 to K. Loré), the Swedish Research Council (2019-01036 and 2020–05829 to K. Loré), the Knut and Alice Wallenberg Foundation through SciLifeLab and Karolinska Institutet (VC-2021-0017 to K. Loré), and the Coalition for Epidemic Preparedness Innovations (CEPI, RRCU2001 to CureVac AG). This research was also supported by intramural faculty salary grants from Karolinska Institutet (to K. Lenart, F.H., and S.O.) and a grant from the China Scholarship Council (to X.Y.).

AUTHOR CONTRIBUTIONS

Conceptualization, K. Lenart, K.S., B.P., and K. Loré; formal analysis, K. Lenart, R.A.C., and K. Loré; funding acquisition, B.P. and K. Loré.; investigation, K. Lenart, F.H., S.O., X.Y., A.C., R.A.C., I.W., and J.H.; methodology, K. Lenart, F.H., and S.O.; resources, S.O.M., E.J., K.S., S.R., B.P., and K. Loré; supervision, K. Loré; visualization, K. Lenart; writing – original draft, K. Lenart and K. Loré; writing – review & editing, all authors.

DECLARATION OF INTERESTS

S.O.M., E.J., K.S., S.R., and B.P. are employees of CureVac AG.

REFERENCES

1. (2022). COVID19 vaccine tracker. Available online at. https://covid19.trackvaccines.org/.
2. Kremsner, P.G., Ahuad Guerrero, R.A., Arana-Arri, E., Aroca Martinez, G.J., Bonten, M., Chandler, R., Corral, G., De Block, E.J.L., Ecker, L., Gabor, J.J., et al. (2022). Efficacy and safety of the CVnCoV SARS-CoV-2 mRNA vaccine candidate in ten countries in Europe and Latin America (HERALD): a randomised, observer-blinded, placebo-controlled, phase 2b/3 trial. Lancet Infect. Dis. *22*, 329–340.
3. Polack, F.P., Thomas, S.J.„ Kitchin, N., Absalon, J., Gurtman, A., Lockhart, S., Perez, J.L., Pérez Marc, G., Moreira, E.D., Zerbini, C., et al. (2020). Safety and efficacy of the BNT162b2 mRNA COVID-19 vaccine. N. Engl. J. Med. *383*, 2603–2615.
4. Baden, L.R., El Sahly, H.M., Essink, B., Kotloff, K., Frey, S., Novak, R., Diemert, D., Spector, S.A., Rouphael, N., Creech, C.B., et al. (2021). Efficacy and safety of the mRNA-1273 SARS-CoV-2 vaccine. N. Engl. J. Med. Overseas. Ed. *384*, 403–416.
5. Kremsner, P.G., Mann, P., Kroidl, A., Leroux-Roels, I., Schindler, C., Gabor, J.J., Schunk, M., Leroux-Roels, G., Bosch, J.J., Fendel, R., et al. (2021). Safety and immunogenicity of an mRNA-lipid nanoparticle vaccine candidate against SARS-CoV-2. Wien Klin. Wochenschr. *133*, 931–941.
6. Gebre, M.S., Rauch, S., Roth, N., Yu, J., Chandrashekar, A., Mercado, N.B., He, X., Liu, J., McMahan, K., Martinot, A., et al. (2022). Optimization of non-coding regions for a non-modified mRNA COVID-19 vaccine. Nature *601*, 410–414.
7. Rauch, S., Gooch, K., Hall, Y., Salguero, F.J., Dennis, M.J., Gleeson, F.V., Harris, D., Ho, C., Humphries, H.E., Longet, S., et al. (2020). mRNA vaccine CVnCoV protects non-human primates from SARS-CoV-2 challenge infection. Preprint at bioRxiv. https://doi.org/10.1101/2020.12.23.424138.
8. van Doremalen, N., Fischer, R.J., Schulz, J.E., Holbrook, M.G., Smith, B.J., Lovaglio, J., Petsch, B., and Munster, V.J. (2021). Immunogenicity of low-dose prime-boost vaccination of mRNA vaccine CV07050101 in non-human primates. Viruses *13*, 1645.
9. Kwissa, M., Nakaya, H.I., Oluoch, H., and Pulendran, B. (2012). Distinct TLR adjuvants differentially stimulate systemic and local innate immune responses in nonhuman primates. Blood *119*, 2044–2055.
10. Thompson, E.A., Ols, S., Miura, K., Rausch, K., Narum, D.L., Spångberg, M., Juraska, M., Wille-Reece, U., Weiner, A., Howard, R.F., et al. (2018). TLR-adjuvanted nanoparticle vaccines differentially influence the quality and longevity of responses to malaria antigen Pfs25. JCI Insight *3*, e120692.
11. Arunachalam, P.S., Scott, M.K.D., Hagan, T., Li, C., Feng, Y., Wimmers, F., Grigoryan, L., Trisal, M., Edara, V.V., Lai, L., et al. (2021). Systems vaccinology of the BNT162b2 mRNA vaccine in humans. Nature *596*, 410–416.
12. Wolz, O.-O., Kays, S.-K., Junker, H., Koch, S.D., Mann, P., Quintini, G., von Eisenhart-Rothe, P., and Oostvogels, L. (2022). A third dose of the COVID-19 vaccine, CVnCoV, increased the neutralizing activity against the SARS-CoV-2 wild-type and Delta variant. Vaccines *10*, 508.
13. Garcia-Beltran, W.F., St Denis, K.J.„ Hoelzemer, A., Lam, E.C., Nitido, A.D., Sheehan, M.L., Berrios, C., Ofoman, O., Chang, C.C., Hauser, B.M., et al. (2022). mRNA-based COVID-19 vaccine boosters induce neutralizing immunity against SARS-CoV-2 Omicron variant. Cell *185*, 457–466.e4.
14. Falsey, A.R., Frenck, R.W., Jr., Walsh, E.E., Kitchin, N., Absalon, J., Gurtman, A., Lockhart, S., Bailey, R., Swanson, K.A., Xu, X., et al. (2021). SARS-CoV-2 neutralization with BNT162b2 vaccine dose 3. N. Engl. J. Med. *385*, 1627–1629.
15. Wratil, P.R., Stern, M., Priller, A., Willmann, A., Almanzar, G., Vogel, E., Feuerherd, M., Cheng, C.-C., Yazici, S., Christa, C., et al. (2022). Three exposures to the spike protein of SARS-CoV-2 by either infection or vaccination elicit superior neutralizing immunity to all variants of concern. Nat. Med. *28*, 496–503.
16. Röltgen, K., Nielsen, S.C.A., Silva, O., Younes, S.F., Zaslavsky, M., Costales, C., Yang, F., Wirz, O.F., Solis, D., Hoh, R.A., et al. (2022). Immune imprinting, breadth of variant recognition, and germinal center response in human SARS-CoV-2 infection and vaccination. Cell *185*, 1025–1040.e14.
17. Corbett, K.S., Flynn, B., Foulds, K.E., Francica, J.R., Boyoglu-Barnum, S., Werner, A.P., Flach, B., O'Connell, S., Bock, K.W., Minai, M., et al. (2020). Evaluation of

the mRNA-1273 vaccine against SARS-CoV-2 in nonhuman primates. N. Engl. J. Med. *383*, 1544–1555.

18. Vogel, A.B., Kanevsky, I., Che, Y., Swanson, K.A., Muik, A., Vormehr, M., Kranz, L.M., Walzer, K.C., Hein, S., Güler, A., et al. (2021). BNT162b vaccines protect rhesus macaques from SARS-CoV-2. Nature *592*, 283–289.

19. Widge, A.T., Rouphael, N.G., Jackson, L.A., Anderson, E.J., Roberts, P.C., Makhene, M., Chappell, J.D., Denison, M.R., Stevens, L.J., Pruijssers, A.J., et al. (2021). Durability of responses after SARS-CoV-2 mRNA-1273 vaccination. N. Engl. J. Med. *384*, 80–82.

20. Sahin, U., Muik, A., Derhovanessian, E., Vogler, I., Kranz, L.M., Vormehr, M., Baum, A., Pascal, K., Quandt, J., Maurus, D., et al. (2020). COVID-19 vaccine BNT162b1 elicits human antibody and T_H1 T cell responses. Nature *586*, 594–599.

21. Corbett, K.S., Nason, M.C., Flach, B., Gagne, M., O'Connell, S., Johnston, T.S., Shah, S.N., Edara, V.V., Floyd, K., Lai, L., et al. (2021). Immune correlates of protection by mRNA-1273 vaccine against SARS-CoV-2 in nonhuman primates. Science *373*, eabj0299.

22. Khoury, D.S., Cromer, D., Reynaldi, A., Schlub, T.E., Wheatley, A.K., Juno, J.A., Subbarao, K., Kent, S.J., Triccas, J.A., and Davenport, M.,P. (2021). Neutralizing antibody levels are highly predictive of immune protection from symptomatic SARS-CoV-2 infection. Nat. Med. *27*, 1205–1211.

23. Cromer, D., Steain, M., Reynaldi, A., Schlub, T.E., Wheatley, A.K., Juno, J.A., Kent, S.J., Triccas, J.A., Khoury, D.S., and Davenport, M.P. (2022). Neutralising antibody titres as predictors of protection against SARS-CoV-2 variants and the impact of boosting: a meta-analysis. Lancet. Microbe *3*, e52–e61.

24. Goel, R.R., Painter, M.M., Apostolidis, S.A., Mathew, D., Meng, W., Rosenfeld, A.M., Lundgreen, K.A., Reynaldi, A., Khoury, D.S., Pattekar, A., et al. (2021). mRNA vaccines induce durable immune memory to SARS-CoV-2 and variants of concern. Science *374*, abm0829.

25. Cagigi, A., Yu, M., Österberg, B., Svensson, J., Falck-Jones, S., Vangeti, S., Åhlberg, E., Azizmohammadi, L., Warnqvist, A., Falck-Jones, R., et al. (2021). Airway antibodies emerge according to COVID-19 severity and wane rapidly but reappear after SARS-CoV-2 vaccination. JCI Insight *6*, e151463.

26. Lederer, K., Bettini, E., Parvathaneni, K., Painter, M.M., Agarwal, D., Lundgreen, K.A., Weirick, M., Muralidharan, K., Castaño, D., Goel, R.R., et al. (2022). Germinal center responses to SARS-CoV-2 mRNA vaccines in healthy and immunocompromised individuals. Cell *185*, 1008–1024.e15.

27. Byrnes, J.R., Zhou, X.X., Lui, I., Elledge, S.K., Glasgow, J.E., Lim, S.A., Loudermilk, R.P., Chiu, C.Y., Wang, T.T., Wilson, M.R., et al. (2020). Competitive SARS-CoV-2 serology reveals most antibodies targeting the spike receptor-binding domain compete for ACE2 binding. mSphere *5*, e00802–e00820.

28. Vázquez Bernat, N., Corcoran, M., Nowak, I., Kaduk, M., Castro Dopico, X., Narang, S., Maisonasse, P., Dereuddre-Bosquet, N., Murrell, B., and Karlsson Hedestam, G.B. (2021). Rhesus and cynomolgus macaque immunoglobulin heavy-chain genotyping yields comprehensive databases of germline VDJ alleles. Immunity *54*, 355–366.e4. e4.

29. Kim, W., Zhou, J.Q., Horvath, S.C., Schmitz, A.J., Sturtz, A.J., Lei, T., Liu, Z., Kalaidina, E., Thapa, M., Alsoussi, W.B., et al. (2022). Germinal centre-driven maturation of B cell response to mRNA vaccination. Nature *604*, 141–145.

30. Dejnirattisai, W., Zhou, D., Ginn, H.M., Duyvesteyn, H.M.E., Supasa, P., Case, J.B., Zhao, Y., Walter, T.S., Mentzer, A.J., Liu, C., et al. (2021). The antigenic anatomy of SARS-CoV-2 receptor binding domain. Cell *184*, 2183–2200.e22.

31. Goel, R.R., Painter, M.M., Lundgreen, K.A., Apostolidis, S.A., Baxter, A.E., Giles, J.R., Mathew, D., Pattekar, A., Reynaldi, A., Khoury, D.S., et al. (2022). Efficient recall of Omicron-reactive B cell memory after a third dose of SARS-CoV-2 mRNA vaccine. Cell *185*, P1875–P1887.e8.

32. Liang, F., Lindgren, G., Lin, A., Thompson, E.A., Ols, S., Röhss, J., John, S., Hassett, K., Yuzhakov, O., Bahl, K., et al. (2017). Efficient targeting and activation of antigen-presenting cells in vivo after modified mRNA vaccine administration in Rhesus macaques. Mol. Ther. *25*, 2635–2647.

33. Lindsay, K.E., Bhosle, S.M., Zurla, C., Beyersdorf, J., Rogers, K.A., Vanover, D., Xiao, P., Araínga, M., Shirreff, L.M., Pitard, B., et al. (2019). Visualization of early events in mRNA vaccine delivery in non-human primates via PET-CT and near-infrared imaging. Nat. Biomed. Eng. *3*, 371–380.

34. Liang, F., Lindgren, G., Sandgren, K.J., Thompson, E.A., Francica, J.R., Seubert, A., De Gregorio, E., Barnett, S., O'Hagan, D.T., Sullivan, N.J., et al. (2017). Vaccine priming is restricted to draining lymph nodes and controlled by adjuvant-mediated antigen uptake. Sci. Transl. Med. *9*, eaal2094.

35. Ols, S., Yang, L., Thompson, E.A., Pushparaj, P., Tran, K., Liang, F., Lin, A., Eriksson, B., Karlsson Hedestam, G.B., Wyatt, R.T., and Loré, K. (2020). Route of vaccine administration alters antigen trafficking but not innate or adaptive immunity. Cell Rep. *30*, 3964–3971.e7.

36. Munro, A.P.S., Janani, L., Cornelius, V., Aley, P.K., Babbage, G., Baxter, D., Bula, M., Cathie, K., Chatterjee, K., Dodd, K., et al. (2021). Safety and immunogenicity of seven COVID-19 vaccines as a third dose (booster) following two doses of ChAdOx1 nCov-19 or BNT162b2 in the UK (COV-BOOST): a blinded, multicentre, randomised, controlled, phase 2 trial. Lancet *398*, 2258–2276.

37. Harvey, W.T., Carabelli, A.M., Jackson, B., Gupta, R.K., Thomson, E.C., Harrison, E.M., Ludden, C., Reeve, R., Rambaut, A., Peacock, S.J., et al. (2021). SARS-CoV-2 variants, spike mutations and immune escape. Nat. Rev. Microbiol. *19*, 409–424.

38. Gagne, M., Corbett, K.S., Flynn, B.J., Foulds, K.E., Wagner, D.A., Andrew, S.F., Todd, J.P.M., Honeycutt, C.C., McCormick, L., Nurmukhambetova, S.T., et al. (2022). Protection from SARS-CoV-2 Delta one year after mRNA-1273 vaccination in rhesus macaques coincides with anamnestic antibody response in the lung. Cell *185*, 113–130.e15.

39. Alberer, M., Gnad-Vogt, U., Hong, H.S., Mehr, K.T., Backert, L., Finak, G., Gottardo, R., Bica, M.A., Garofano, A., Koch, S.D., et al. (2017). Safety and immunogenicity of a mRNA rabies vaccine in healthy adults: an open-label, non-randomised, prospective, first-in-human phase 1 clinical trial. Lancet *390*, 1511–1520.

40. Aldrich, C., Leroux-Roels, I., Huang, K.B., Bica, M.A., Loeliger, E., Schoenborn-Kellenberger, O., Walz, L., Leroux-Roels, G., von Sonnenburg, F., and Oostvogels, L. (2021). Proof-of-concept of a low-dose unmodified mRNA-based rabies vaccine formulated with lipid nanoparticles in human volunteers: a phase 1 trial. Vaccine *39*, 1310–1318.

41. Cagigi, A., and Loré, K. (2021). Immune responses induced by mRNA vaccination in mice, monkeys and humans. Vaccines (Basel) *9*, 61.

42. Lindgren, G., Ols, S., Liang, F., Thompson, E.A., Lin, A., Hellgren, F., Bahl, K., John, S., Yuzhakov, O., Hassett, K.J., et al. (2019). Induction of robust B cell responses after influenza mRNA vaccination is accompanied by circulating hemagglutinin-specific ICOS+ PD-1+ CXCR3+ T follicular helper cells. Front. Immunol. *10*, 614.

43. Gujer, C., Sandgren, K.J., Douagi, I., Adams, W.C., Sundling, C., Smed-Sörensen, A., Seder, R.A., Karlsson Hedestam, G.B., and Loré, K. (2011). IFN-alpha produced by human plasmacytoid dendritic cells enhances T cell-dependent naive B cell differentiation. J. Leukoc. Biol. *89*, 811–821.

44. Le Bon, A., Schiavoni, G., D'Agostino, G., Gresser, I., Belardelli, F., and Tough, D.F. (2001). Type i interferons potently enhance humoral immunity and can promote isotype switching by stimulating dendritic cells in vivo. Immunity *14*, 461–470.

45. Johnson, T.R., Rao, S., Seder, R.A., Chen, M., and Graham, B.S. (2009). TLR9 agonist, but not TLR7/8, functions as an adjuvant to diminish FI-RSV vaccine-enhanced disease, while either agonist used as therapy during primary RSV infection increases disease severity. Vaccine *27*, 3045–3052.

46. Francica, J.R., Sheng, Z., Zhang, Z., Nishimura, Y., Shingai, M., Ramesh, A., Keele, B.F., Schmidt, S.D., Flynn, B.J., Darko, S., et al. (2015). Analysis of immunoglobulin transcripts and hypermutation following SHIV(AD8) infection and protein-plus-adjuvant immunization. Nat. Commun. *6*, 6565.

47. Shah, J.A., Darrah, P.A., Ambrozak, D.R., Turon, T.N., Mendez, S., Kirman, J., Wu, C.-Y., Glaichenhaus, N., Seder, R.A., et al. (2003). Dendritic cells are responsible for the capacity of CpG oligodeoxynucleotides to act as an adjuvant for protective vaccine immunity against Leishmania major in mice. J. Exp. Med. *198*, 281–291.

48. Tewari, K., Flynn, B.J., Boscardin, S.B., Kastenmueller, K., Salazar, A.M., Anderson, C.A., Soundarapandian, V., Ahumada, A., Keler, T., Hoffman, S.L., et al. (2010). Poly(I:C) is an effective adjuvant for antibody and multi-functional CD4+ T cell responses to Plasmodium falciparum circumsporozoite protein (CSP) and αDEC-CSP in non human primates. Vaccine *28*, 7256–7266.

49. Bergamaschi, C., Terpos, E., Rosati, M., Angel, M., Bear, J., Stellas, D., Karaliota, S., Apostolakou, F., Bagratuni, T., Patseas, D., et al. (2021). Systemic IL-15, IFN-γ,

and IP-10/CXCL10 signature associated with effective immune response to SARS-CoV-2 in BNT162b2 mRNA vaccine recipients. Cell Rep. *36*, 109504.

50. Jakubzick, C.V., Randolph, G.J., and Henson, P.M. (2017). Monocyte differentiation and antigen-presenting functions. Nat. Rev. Immunol. *17*, 349–362.

51. Kwissa, M., Nakaya, H.I., Onlamoon, N., Wrammert, J., Villinger, F., Perng, G.C., Yoksan, S., Pattanapanyasat, K., Chokephaibulkit, K., Ahmed, R., and Pulendran, B. (2014). Dengue virus infection induces expansion of a CD14(+)CD16(+) monocyte population that stimulates plasmablast differentiation. Cell Host Microbe *16*, 115–127.

52. Zhu, H., Hu, F., Sun, X., Zhang, X., Zhu, L., Liu, X., Li, X., Xu, L., Shi, L., Gan, Y., and Su, Y. (2016). CD16 + monocyte subset was enriched and functionally exacerbated in driving T-cell activation and B-cell response in systemic lupus erythematosus. Front. Immunol. *7*, 512.

53. Glaros, V., Rauschmeier, R., Artemov, A.V., Reinhardt, A., Ols, S., Emmanouilidi, A., Gustafsson, C., You, Y., Mirabello, C., Björklund, Å.K., et al. (2021). Limited access to antigen drives generation of early B cell memory while restraining the plasmablast response. Immunity *54*, 2005–2023.e10.

54. Voss, W.N., Hou, Y.J., Johnson, N.V., Delidakis, G., Kim, J.E., Javanmardi, K., Horton, A.P., Bartzoka, F., Paresi, C.J., Tanno, Y., et al. (2021). Prevalent, protective, and convergent IgG recognition of SARS-CoV-2 non-RBD spike epitopes. Science *372*, 1108–1112.

55. Amanat, F., Thapa, M., Lei, T., Ahmed, S.M.S., Adelsberg, D.C., Carreño, J.M., Strohmeier, S., Schmitz, A.J., Zafar, S., Zhou, J.Q., et al. (2021). SARS-CoV-2 mRNA vaccination induces functionally diverse antibodies to NTD, RBD, and S2. Cell *184*, 3936–3948.e10. e10.

56. Beaudoin-Bussières, G., Chen, Y., Ullah, I., Prévost, J., Tolbert, W.D., Symmes, K., Ding, S., Benlarbi, M., Gong, S.Y., Tauzin, A., et al. (2022). A Fc-enhanced NTD-binding non-neutralizing antibody delays virus spread and synergizes with a nAb to protect mice from lethal SARS-CoV-2 infection. Cell Rep. *38*, 110368.

57. Bahnan, W., Wrighton, S., Sundwall, M., Bläckberg, A., Larsson, O., Höglund, U., Khakzad, H., Godzwon, M., Walle, M., Elder, E., et al. (2021). Spike-dependent opsonization indicates both dose-dependent inhibition of phagocytosis and that non-neutralizing antibodies can confer protection to SARS-CoV-2. Front. Immunol. *12*, 808932.

58. Feng, S., Phillips, D.J., White, T., Sayal, H., Aley, P.K., Bibi, S., Dold, C., Fuskova, M., Gilbert, S.C., Hirsch, I., et al. (2021). Correlates of protection against symptomatic and asymptomatic SARS-CoV-2 infection. Nat. Med. *27*, 2032–2040.

59. Sáez-Llorens, X., Lanata, C., Aranguren, E., Celis, C.R., Cornejo, R., DeAntonio, R., Ecker, L., Garrido, D., Gil, A.I., Gonzales, M., et al. (2022). Safety and immunogenicity of mRNA-LNP COVID-19 vaccine CVnCoV in Latin American adults: a phase 2 randomized study, Vaccine X In press. https://doi.org/10.1016/j.jvacx.2022.100189.

60. Griesbeck, O., Baird, G.S., Campbell, R.E., Zacharias, D.A., and Tsien, R.Y. (2001). Reducing the environmental sensitivity of yellow fluorescent protein. J. Biol. Chem. *276*, 29188–29194.

61. Yuan, M., Wu, N.C., Zhu, X., Lee, C.-C.D., So, R.T.Y., Lv, H., Mok, C.K.P., and Wilson, I.A. (2020). A highly conserved cryptic epitope in the receptor binding domains of SARS-CoV-2 and SARS-CoV. Science *368*, 630–633.

62. Wu, Y., Wang, F., Shen, C., Peng, W., Li, D., Zhao, C., Li, Z., Li, S., Bi, Y., Yang, Y., et al. (2020). A noncompeting pair of human neutralizing antibodies block COVID-19 virus binding to its receptor ACE2. Science *368*, 1274–1278.

63. Robbiani, D.F., Gaebler, C., Muecksch, F., Lorenzi, J.C.C., Wang, Z., Cho, A., Agudelo, M., Barnes, C.O., Gazumyan, A., Finkin, S., et al. (2020). Convergent antibody responses to SARS-CoV-2 in convalescent individuals. Nature *584*, 437–442.

64. Pinto, D., Park, Y.-J., Beltramello, M., Walls, A.C., Tortorici, M.A., Bianchi, S., Jaconi, S., Culap, K., Zatta, F., De Marco, A., et al. (2020). Cross-neutralization of SARS-CoV-2 by a human monoclonal SARS-CoV antibody. Nature *583*, 290–295.

65. Tortorici, M.A., Beltramello, M., Lempp, F.A., Pinto, D., Dang, H.V., Rosen, L.E., McCallum, M., Bowen, J., Minola, A., Jaconi, S., et al. (2020). Ultrapotent human antibodies protect against SARS-CoV-2 challenge via multiple mechanisms. Science *370*, 950–957.

66. McCallum, M., De Marco, A., Lempp, F.A., Tortorici, M.A., Pinto, D., Walls, A.C., Beltramello, M., Chen, A., Liu, Z., Zatta, F., et al. (2021). N-terminal domain antigenic mapping reveals a site of vulnerability for SARS-CoV-2. Cell *184*, 2332–2347.e16.

67. Brouwer, P.J.M., Caniels, T.G., van der Straten, K., Snitselaar, J.L., Aldon, Y., Bangaru, S., Torres, J.L., Okba, N.M.A., Claireaux, M., Kerster, G., et al. (2020). Potent neutralizing antibodies from COVID-19 patients define multiple targets of vulnerability. Science *369*, 643–650.

68. Manenti, A., Maggetti, M., Casa, E., Martinuzzi, D., Torelli, A., Trombetta, C.M., Marchi, S., and Montomoli, E. (2020). Evaluation of SARS-CoV-2 neutralizing antibodies using a CPE-based colorimetric live virus micro-neutralization assay in human serum samples. J. Med. Virol. *92*, 2096–2104.

69. Bewley, K.R., Coombes, N.S., Gagnon, L., McInroy, L., Baker, N., Shaik, I., St-Jean, J.R., St-Amant, N., Buttigieg, K.R., Humphries, H.E., et al. (2021). Quantification of SARS-CoV-2 neutralizing antibody by wild-type plaque reduction neutralization, microneutralization and pseudotyped virus neutralization assays. Nat. Protoc. *16*, 3114–3140.

70. Sundling, C., Phad, G., Douagi, I., Navis, M., and Karlsson Hedestam, G.B. (2012). Isolation of antibody V(D)J sequences from single cell sorted rhesus macaque B cells. J. Immunol. Methods *386*, 85–93.

71. Corcoran, M.M., Phad, G.E., Vázquez Bernat, N., Stahl-Hennig, C., Sumida, N., Persson, M.A.A., Martin, M., and Karlsson Hedestam, G.B. (2016). Production of individualized V gene databases reveals high levels of immunoglobulin genetic diversity. Nat. Commun. *7*, 13642.

72. Phad, G.E., Pushparaj, P., Tran, K., Dubrovskaya, V., Àdori, M., Martinez-Murillo, P., Vázquez Bernat, N., Singh, S., Dionne, G., O'Dell, S., et al. (2020). Extensive dissemination and intraclonal maturation of HIV Env vaccine-induced B cell responses. J. Exp. Med. *217*, e20191155.

73. Li, D., Edwards, R.J., Manne, K., Martinez, D.R., Schäfer, A., Alam, S.M., Wiehe, K., Lu, X., Parks, R., Sutherland, L.L., et al. (2021). In vitro and in vivo functions of SARS-CoV-2 infection-enhancing and neutralizing antibodies. Cell *184*, 4203–4219.e32.

OMTM, Volume *27*

Supplemental information

A third dose of the unmodified COVID-19 mRNA vaccine CVnCoV enhances quality and quantity of immune responses

Klara Lenart, Fredrika Hellgren, Sebastian Ols, Xianglei Yan, Alberto Cagigi, Rodrigo Arcoverde Cerveira, Inga Winge, Jakub Hanczak, Stefan O. Mueller, Edith Jasny, Kim Schwendt, Susanne Rauch, Benjamin Petsch, and Karin Loré

SUPPLEMENTAL MATERIALS

SUPPLEMENTAL FIGURES

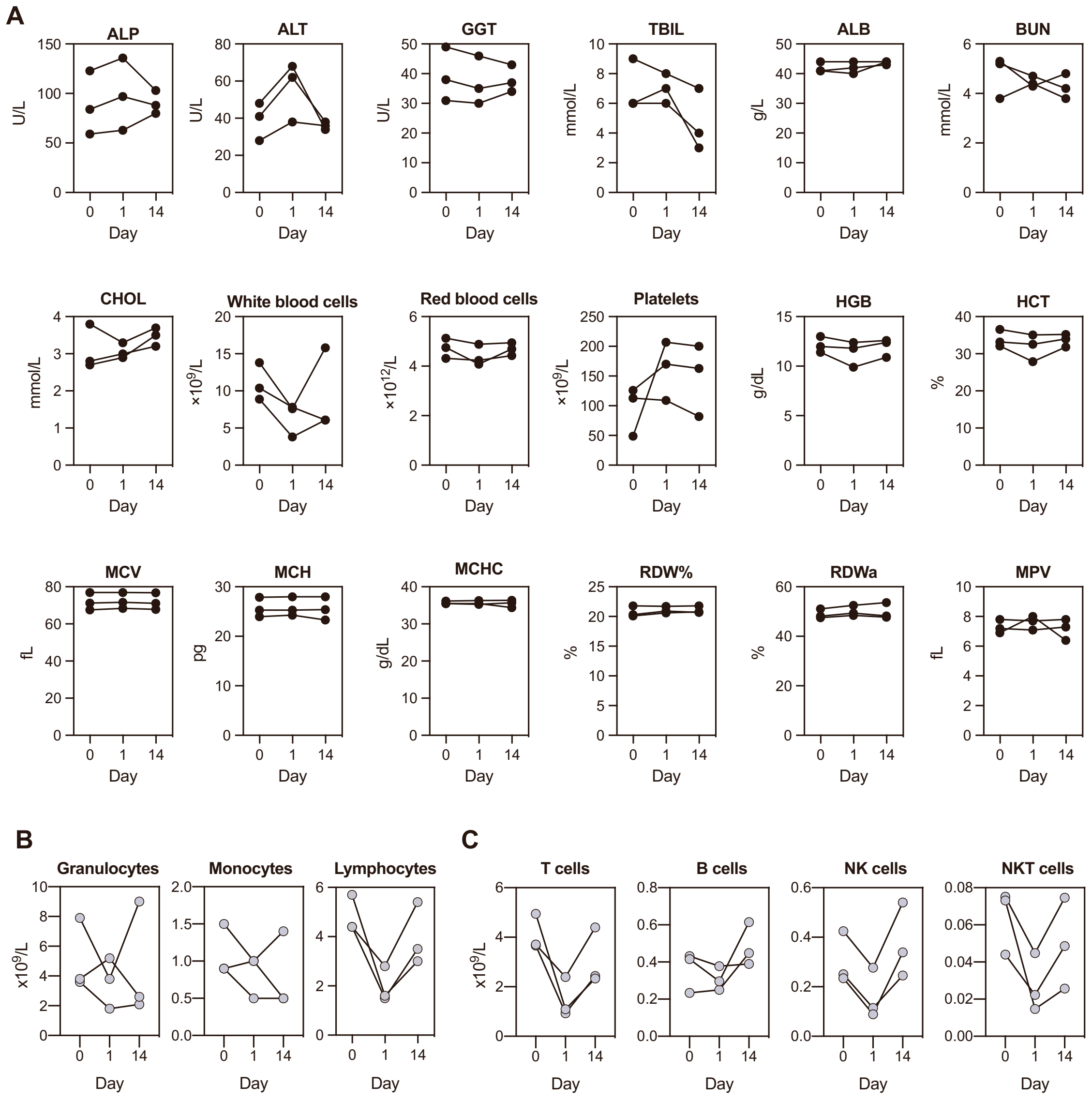

Figure S1: **Safety and innate data after prime mRNA immunization.** (A) Clinical chemistry and hematology analyses at 0, 1 and 14 days after prime immunization. (B) Complete blood counts (CBCs) performed at 0, 1 and 14 days after prime immunization. (C) Immune cell subsets quantified by flow cytometry and normalized to lymphocyte CBC data at days 0, 1 and 14 after prime immunization. ALP = Alkaline Phosphatase, ALT = Alanine Aminotransferase, GGT = Gamma-Glutamyl Transferase, TBIL = Total Bilirubin, ALB = Albumin, BUN = Blood Urea Nitrogen, CHOL = Cholesterol, HGB = Hemoglobin, HCT = Hematocrit, MCV = Mean Corpuscular Volume, MCH = Mean Corpuscular Hemoglobin, MCHC = Mean Corpuscular Hemoglobin Concentration, RDW% = Red Cell Distribution, RDWa = Red Cell Distribution (Absolute), MPV = Mean Platelet Volume.

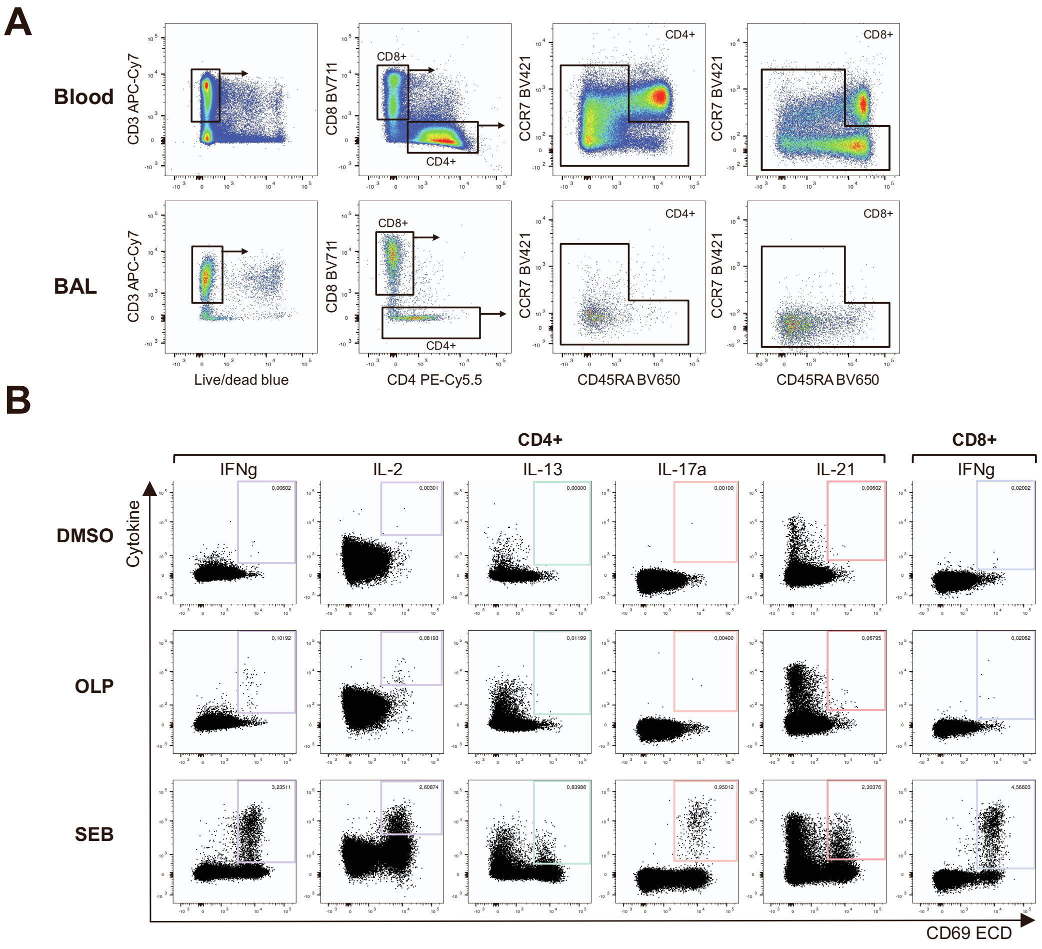

Figure S2. (A) Gating strategy used to define CD4+ and CD8+ T cells in blood and BAL. (B) Definition of responding antigen-specific T cells by intracellular cytokine staining.

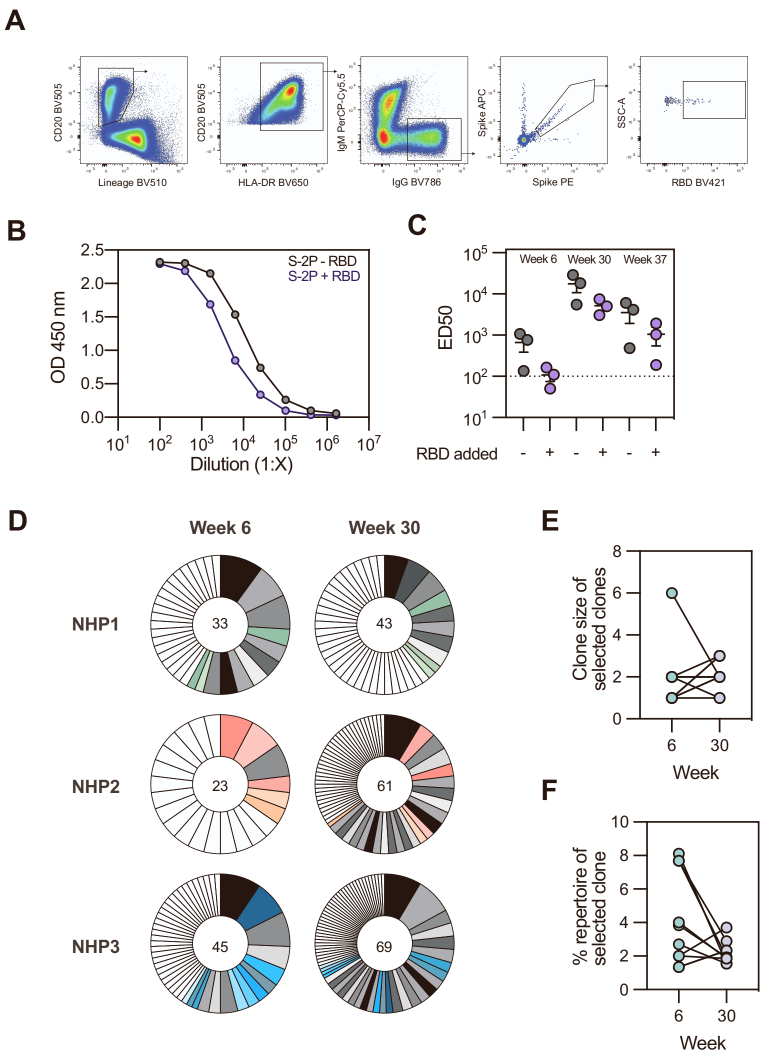

Figure S3. (A) Gating strategy used to define antigen-specific memory B cells. Lineage channel contains CD3, CD11c, CD14, CD16 and CD123. (B, C) ELISA curves (B) and ED50 values (C) of NHP sera with and without soluble RBD as competitor, used to calculate proportion of RBD-binding antibodies. (D) Clonotyping of sorted Spike-specific memory B cells. Each pie chart represents the sequenced repertoire in a NHP at a selected timepoint. The number in the middle indicates number of clones. Pie slice size is proportional to the clone expansion at a given timepoint. Colored pie slices represent clones detected at both analyzed timepoints. (E, F) Sizes of clones, from which selected mAbs were derived from, plotted as absolute number of sequences in the clone (E) and as percentage of repertoire sequenced (F).

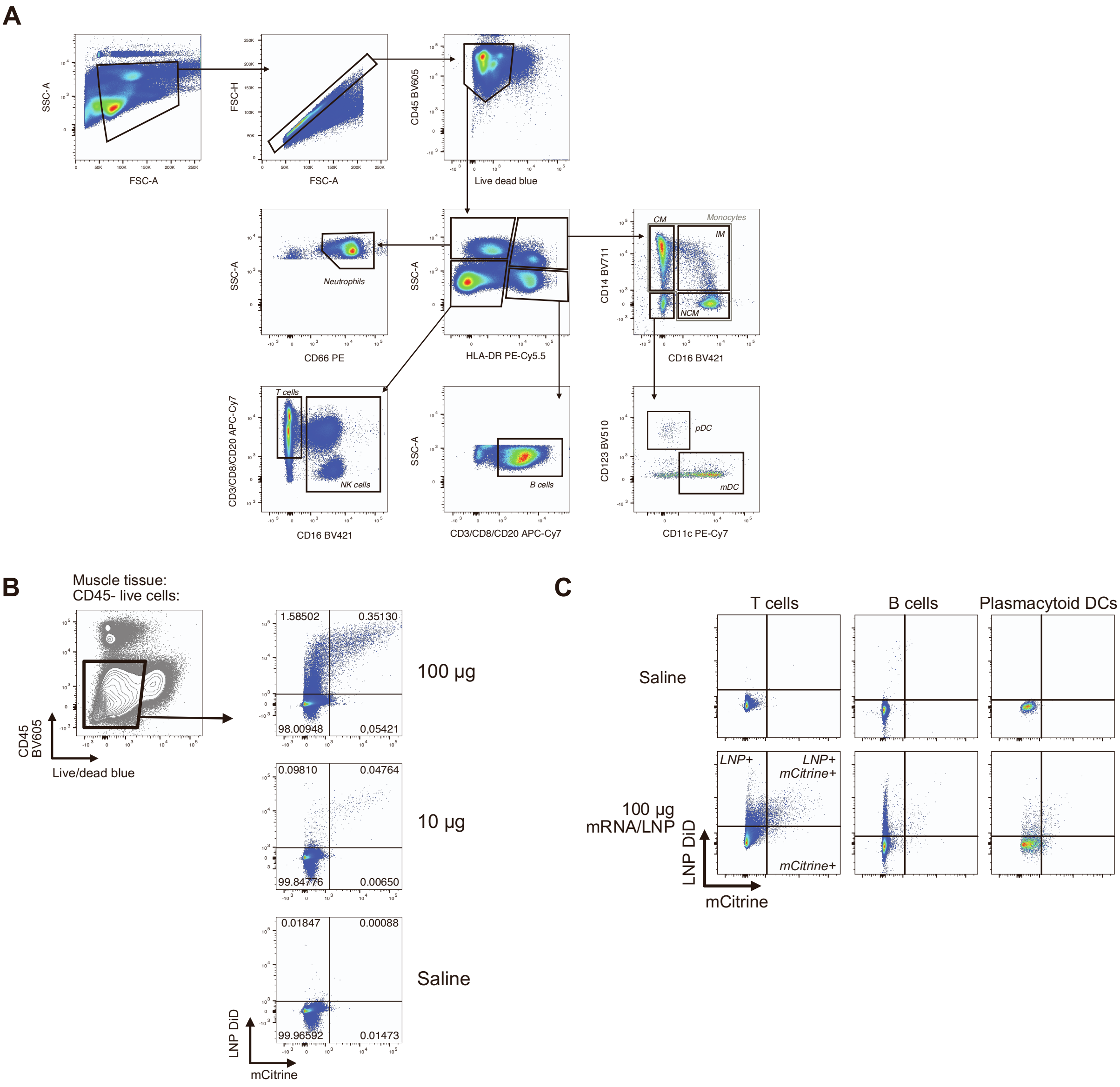

Figure S4. (A) Gating strategy used to define immune cell subsets in blood and tissues after mRNA vaccine immunization in biodistribution experiments. (B) LNP uptake and mRNA vaccine-derived protein expression in non-immune cells at site of injection (muscle tissue). (C) Representative flow cytometry plots of LNP DiD and mCitrine signal in draining lymph nodes by cell subset (T cells, B cell and plasmacytoid DCs) from saline- and vaccine-injected sites.

SUPPLEMENTAL TABLES

Table S1: Innate immunophenotyping antibody panel

Antibody	Clone	Manufacturer
CD40-FITC	5C3	Biolegend
NKg2a-PE	Z199	Beckman Coulter
CD80-BV421	L307.4	BD
CCR7-PE-Dazzle 594	G043H7	Biolegend
CD123-PerCp-Cy5.5	7G3	BD
CD3-APC-Cy7	SP34-2	BD
CD66-APC	TET2	Miltenyi
CD70-BV786	Ki-24	BD
HLA-DR-BV650	L243	Biolegend
CD11c-PE-Cy7	3.9	Biolegend
CD16-AF700	38G	BD
CD20-BV605	2H7	Biolegend
CD14-BV510	M5E2	Biolegend

Table S2: T cell ICS staining antibody panel (*Intracellular staining)

Antibody	Clone	Manufacturer
CD103-FITC	2G5	Beckman
CCR7-BV421	G043H7	Biolegend
CD8a-BV711	RPA-T8	Biolegend
CD4-PE-Cy55	S3.5	Invitrogen
CD45RA-BV650	5H9	BD
*IL-21-AF647	3A3-N2.1	BD
*IL-13-PE	JES10-5A2	BD
*IL-2-BV605	MQ1-17H12	BD
*IL-17A-BV785	BL168	Biolegend
*CD69-ECD	TP1.55.3	Beckman
*CD3-APC-Cy7	SP34.2	BD
*IFNg-AF700	B27	Biolegend

Table S3: Memory B cell antibody panel

Antibody	Clone	Manufacturer
7AAD	NA	Thermo Fisher
IgM-PerCP-Cy5.5	G20-127	BD
CD3-BV510	SP34-2	BD
CD123-BV510	6H6	Biolegend
CD11c-BV510	3.9	Biolegend
CD16-BV510	3G8	BD
HLA-DR-BV650	L243	Biolegend
IgG-BV786	G18-145	BD
CD20-BV605	2H7	Biolegend
CD14-BV510	M5E2	Biolegend

Table S4: Biodistribution antibody panel (*NHP samples only)

Antibody	Clone	Manufacturer
CD66abce-PE	TET2	Miltenyi
CD11c-PE-Cy7	3.9	Biolegend
CD14-BV711	M5E2	Biolegend
CD123-BV510	6H6	Biolegend
CD3-APC-Cy7	SP34-2	BD
CD20-APC-Cy7	L27	BD
CD8-APC-Cy7	RPA-T8	Biolegend
CD80-BV650	L307.4	BD
*CD45-BV605	D058-1283	BD
HLA-DR-PE-Cy55	Tu36	Life Tech
CD16-BV421	3G8	Biolegend
